# Oxygen attachment dissociation (OAD) MS/MS in the identification of positional isomers of dysregulated lipids detected in an ethanol exposure metabolomics study in mice

**DOI:** 10.1007/s11306-025-02282-8

**Published:** 2025-07-01

**Authors:** Emily G. Armitage, Alan Barnes, Olga Deda, Christina Virgiliou, Neil J. Loftus, Helen Gika, Ian D. Wilson

**Affiliations:** 1 Shimadzu Corporation, Manchester, M17 1GP UK; 2 Department of Medicine, Aristotle University of Thessaloniki, Thessaloniki, 54124 Greece; 3 BiOMIC_AUTh, Centre for Interdisciplinary Research and Innovation (CIRI-AUTH), Balkan Centre, Thessaloniki, 57001 Greece; 4 Department of Chemical Engineering, Aristotle University of Thessaloniki, Thessaloniki, 54124 Greece; 5 Computational & Systems Medicine, Department of Metabolism, Digestion and Reproduction, Imperial College, Burlington Danes Building, Du Cane Road, London, W12 0NN UK

**Keywords:** Lipidomics, Carbon-carbon double bonds, Ethanol, Tissues, Oxygen attachment dissociation, MS/MS

## Abstract

**Introduction:**

In metabolic profiling studies the structural characterisation of lipids requires the identification of the head group, carbon number and the position(s) of carbon-carbon double bonds (C = C). Locating the position of double bonds is vital since minor structural differences between positional isomers can alter a lipid’s biochemical function.

**Objectives:**

Oxygen Attachment Dissociation (OAD) is a novel fragmentation technology that enables the localisation of C = C double bonds in lipids. To evaluate its use in the structural characterisation of lipids, OAD has been applied in a discovery-based untargeted analysis of the metabolic impact of acute ethanol exposure in a mouse model.

**Methods:**

UHPLC-OAD-MS/MS was used to enhance the identification of lipids found to be significantly altered by acute ethanol exposure in the gut, liver and pancreas tissues of male C57BL/6 mice receiving a Lieber-DeCarli liquid diet either containing 5% ethanol or an isocaloric control diet. Tissue extracts were analysed using untargeted UHPLC-DIA-MS/MS; UHPLC-OAD-MS/MS analysis was performed to further annotate lipids that were significantly increased or diminished in the animals exposed to ethanol.

**Results:**

UHPLC-DIA-MS/MS analysis of gut, liver and pancreas tissue revealed 101 lipids that were significantly increased or diminished in ethanol treated mice. Of the included 83 unsaturated lipids detected, UHPLC-OAD-MS/MS enabled the localisation of C = C double bonds in 61, including isomers indistinguishable by MS/MS with collision induced dissociation.

**Conclusions:**

The results demonstrate the value of OAD-MS/MS in enhancing lipid identification. The resulting improvement may enable better understanding of the underlying biochemistry in the response of mice to exposure to ethanol.

**Supplementary Information:**

The online version contains supplementary material available at 10.1007/s11306-025-02282-8.

## Introduction

In a typical untargeted metabolomics workflow, metabolic phenotypes of the tissues, cells or biofluids are acquired using advanced analytical techniques to access the metabolic perturbations. High resolution accurate mass spectrometry is commonly employed to analyse biological samples to generate metabolic profiles (metabotyping) and discover metabolite and lipid markers of significance in different sample groups. The identification of differentiating metabolites is, however, a major bottleneck with comparison against authentic standards, using the same analytical method on the same instrument, providing the greatest confidence in identifying significant compounds (Metabolomics Standards Initiative (MSI) level 1 (Spicer et al. 2017; Sumner et al., 2007). However, the limited availability, stability and cost of standards means that many metabolites and lipids can only be annotated and reported to MSI level 2– comparison of experimental data to literature or external laboratory data. This is particularly the case for lipids, where the diversity of potentially relevant variants can be too great to support authentic standard analysis for every lipid in every study. There are now more than 48,000 lipid structures in the LIPID MAPS database and amongst untargeted metabolomics studies, lipids represent more than 60% of all compounds detected and reported (Conroy et al., 2024).

One way to increase the reporting confidence for lipids is to identify additional structural information that can distinguish different isoforms. The structural characterisation of lipids requires the determination of the head group, length of carbon chains and the number and position of double bonds within them. Collision induced dissociation (CID) is useful to determine lipid class and chain length, however identifying carbon-carbon (C = C) double bond position(s) presents a major challenge in unsaturated lipid characterisation. Minor structural differences between positional isomers can markedly change the biological function of a lipid (Ekroos et al., 2003; Kelley et al., 2007; Kyle et al., 2016; Wang et al., 2023). Therefore, locating double bond position(s) in lipids could potentially enhance our understanding of their biological roles.

Several analytical techniques have been developed for the purpose of resolving double bond positions in lipids including chemical derivatization (Feng et al. 2019; Hirtzel et al. 2023; Xie and Xia 2019; Zhang et al. 2019), electron impact excitation of ions from organics (Baba, Campbell, Blanc, et al. 2016; Baba, Campbell, Le Blanc, et al. 2016), oxygen attachment dissociation (OAD)(Takahashi et al. 2018, 2020), ozone-induced dissociation (Barrientos et al. 2017; Marshall et al. 2019; Thomas et al. 2008), radical-directed dissociation (Pham et al. 2012) and ultraviolet photodissociation (Ryan et al. 2017; Williams et al. 2017). OAD enables the investigator to obtain diagnostic fragments for the characterisation of C = C double bonds at higher efficiency in both positive and negative ion modes. The mechanism, as previously described (Takahashi et al. 2018, 2020; Uchino et al. 2022), involves the generation of O/OH• radicals by microwave discharge of water vapour which are introduced into the collision cell of the MS/MS instrument via an inductively coupled plasma (ICP) through a quartz tube. Neutral radicals interact with precursor ions to specifically oxidise/dissociate the double bonds between the carbons. With a careful balance of collision cell gas pressure and collision energy spread, spectra can be acquired with simultaneous OAD-MS/MS and CID-MS/MS (in either positive or negative ion mode) on a high resolution QTOF instrument to generate sufficient information to identify lipids to the structural level.

To highlight the value structural characterisation of lipids can bring to discovery-based untargeted metabolomics, the OAD technique was applied here in a study aimed at exploring the metabolic effects of acute ethanol exposure to mice. Previously we found that ethanol treatment produced specific changes in the metabotypes of the brain and excreta (urine and faeces) of these mice depending upon on the type and duration of exposure (Deda et al., 2019), whereas in other similar studies from our group metabolic perturbations were noted in plasma, urine (Gika et al., 2012) and liver (Loftus et al., 2011). Acute ethanol exposure in the brain caused reductions in nucleosides, fatty acids, and phospholipids, alongside disrupted purine metabolism. Acute exposure increased the relative amounts of neurotransmitters such as glutamate and GABA, while reductions in galactosylceramides were seen, possibly indicating myelin damage (Deda et al., 2020). Perturbed pathways observed in excreta included nitrogen and amino acid metabolism. Biomarkers such as hydroxyphenyllactic acid and indolelactic acid, associated with tyrosine and tryptophan metabolism, were identified as potential indicators of ethanol-induced dysbiosis (Deda et al., 2019).

Here we have analysed tissue extracts from three further organs (liver, gut and pancreas) from these mice using UHPLC-OAD-MS/MS to better characterise organ-specific changes in the lipid profiles of gut, liver and pancreas of mice in response to ethanol.

##  Materials and methods

### Reagents and materials

Methanol and isopropanol used in sample preparation were of LC-MS grade and purchased from Sigma Aldrich (St. Louis, USA and Merck, Darmstadt, Germany). Ultrapure Water (18.2 MΩ cm) was obtained by a Milli-Q purification system (Merck Darmstadt, Germany). For LC-MS/MS analysis, acetonitrile and water (both LC-MS grade) were purchased from Sigma Aldrich.

### Animal experiment

This study used a short-term model (Deda et al., 2019, 2020) to simulate acute ethanol exposure in 28-week-old male C57BL/6 mice (*n* = 16). Over an 11-day period, the mice were divided into two groups: an ethanol-treated group (*n* = 8) and a control group (*n* = 8). The ethanol group received a Lieber-DeCarli liquid diet (DeCarli & Lieber, 1967) containing 5% extra pure ethanol, available ad libitum, while the control group was fed with an isocaloric diet balanced by adding maltose dextrin. To intensify the ethanol exposure, the treated mice received two acute doses of 25% ethanol by oral gavage on the 5th and 11th days. The final dose was administered six hours before sacrifice.

The mice were housed in groups of four per cage under controlled conditions; temperature (22–25 °C), humidity (50%), 12-hour light/dark cycle and health status were monitored weekly. Murine tissues were collected post-mortem, rinsed with saline, snap-frozen in liquid nitrogen, and stored at − 80 °C for later analysis.

The study protocol followed ethical guidelines from the Veterinary Medicine School of Aristotle University of Thessaloniki, in line with European Directive 2010/63 and relevant national regulations (N. 2015/1992, ΠΔ 56/20130) Every effort has been made to present all data in accordance with the ARRIVE guidelines (NC3Rs).

### Sample preparation

Tissues were extracted in methanol: isopropanol: water (3:3:1, v/v/v) at a ratio of 1:3 mg tissue/µL solvent, then homogenised in solution (VWR Bead Mill Max). Following homogenisation, all samples were centrifuged at 8000 × g for 15 min and supernatants collected (150 µL for pancreas and 500 µL for gut and liver). Supernatants were evaporated to dryness under nitrogen and stored at −80 °C until analysis. Dried extracts were reconstituted in methanol using 150 µL for pancreas and 500 µL for gut and liver. Samples were vortexed and then shaken vigorously for 45 min before being chilled on ice for 15 min. Finally, samples were centrifuged at 16,000 × g for 15 min and supernatants transferred to fresh Eppendorf tubes. Preparation of quality control (QC) samples was performed for assessing the quality of the acquired data (Kirwan et al., 2022). For this, 25 µL aliquots were taken from each sample to form tissue-specific (phenotypic) pooled QCs (400 µL per tissue). A combined pooled QC was prepared by taking 100 µL aliquots of each of these phenotypic QCs. All samples including QCs were prepared in LC vials at a dilution of 1 in 20 (10 µL sample in 190 µL methanol or 15 µL QC in 285 µL methanol) for untargeted UHPLC-MS/MS analysis. For UHPLC-OAD-MS/MS analysis, tissue QCs were prepared at a dilution of 1 in 2 in methanol.

### Untargeted LC-MS analysis

#### UHPLC-DIA-MS/MS

Samples were first analysed in three separate batches for liver, gut and pancreas in positive and negative electrospray ionisation (ESI) modes. Each batch started (and ended) with a solvent blank sample then nine injections of the tissue specific (phenotypic) QC were used to equilibrate and stabilise the system, also acting as a system suitability test. Once stabilised the analysis was started with an injection of the pooled QC, then the tissue specific QC and then the samples in randomised order with one of each of the QCs analysed between every five samples. The UHPLC-MS/MS system comprised of a Nexera X2 LC coupled to a LCMS-9030 Q-TOF high resolution accurate mass MS system (Shimadzu Corporation, Kyoto, Japan). Chromatographic separations were performed using an Acquity UHPLC BEH C18 column (1.7 μm, 2.1 × 100 mm, Waters Ltd., Elstree, UK). Samples were maintained at 4 °C and 0.5 µL aliquots were injected onto the column. The chromatographic system used a binary gradient of Solvent A (water with 0.1% formic acid) and Solvent B (acetonitrile with 0.1% formic acid) at a flow rate 0.4 mL/min. The initial gradient conditions were 2% B for 1 min followed by a linear gradient to 40% B for 2 min, then a step curvy-linear gradient up to 90% B at 25 min followed by a linear gradient to 100% B at 27.5 min. These conditions were held for 3.5 min, before returning to the initial conditions of 2% B for 4 min for column re-equilibration. This resulted in a total analysis time per sample of 35 min. Data were acquired using a single method with MS and data independent acquisition (DIA) MS/MS analysis. The method acquired a single TOF MS scan (*m/z* 100–1000) followed by 45 CID-MS/MS mass scans over a mass range of *m/z* 40-1000; each MS/MS mass scan had a precursor isolation width of 20 Da and a collision energy spread of 5–55 V resulting in a cycle time of 1 s. This allowed collection of fragmentation data of all masses in the spectra across the entire LC gradient. The following MS parameters were used: ion source temperature 300 °C; heated capillary temperature 250 °C; heat block temperature 400 °C; electrospray voltage 1.5 kV (positive ESI) or -3.0 kV (negative ESI); electrospray nebulization gas flow 3 L/min; drying gas flow 15 L/min; CID gas pressure 230 kPa. Mass calibration was performed externally using a sodium iodide solution (400 ppm in methanol) from *m/z* 50-1000. Data acquisition was performed using LabSolutions software (Shimadzu Corporation, Kyoto, Japan).

#### LC-DDA-MS/MS

To support subsequent metabolite identification phenotypic QCs were analysed using data dependent acquisition (DDA) MS/MS data in both positive and negative ESI, using the same LC conditions as described above. The DDA MS/MS method acquired a single TOF MS scan (*m/z* 100–1000) followed by 18 MS/MS mass scans over a mass range of *m/z* 40-1000 within a 1 s cycle. Precursors for MS/MS were selected by intensity detected in the TOF MS scan and three MS/MS spectra were collected for each precursor. Isotopes and ions of other charge states, related to the precursors, were excluded as well as the precursor itself for three seconds after being targeted. All other MS parameters were consistent with the UHPLC-DIA-MS/MS method described above.

#### UHPLC- OAD-MS/MS

The phenotypic tissue QC samples were analysed by OAD-MS/MS to structurally characterise lipids found to be significantly differentiated in ethanol treated mouse tissues compared to healthy controls. The UHPLC-MS/MS analysis was performed as described above using a Nexera X2 LC coupled to a LCMS-9050 Q-TOF high resolution accurate mass MS system with the OAD Radical Source I (Shimadzu Corporation, Kyoto, Japan). MS detection was performed using a single method for MS and DDA-MS/MS analysis. Data acquisition considered positive and negative ESI; each method acquired a single TOF MS scan (*m/z* 60-1250) followed by three MS/MS mass scans over a mass range of *m/z* 40-1250 with collision energy spread of 6–30 V within a 1 s cycle. Precursors for MS/MS were selected by intensity detected in the TOF MS scan and three MS/MS spectra were collected for each precursor. Positive ESI mode used unit resolution (0.8 Da) in the quadrupole; isotopes and ions of other charge states related to the precursors were excluded as well as a precursor itself for three seconds after being targeted whilst negative ESI used low resolution (3 Da) in the quadrupole. The MS parameters were the same as described for UHPLC-MS/MS analysis, except for the interface voltage for positive ESI which was set to 4.0 kV and the CID gas pressure which was set to 17 kPa for both positive and negative ESI acquisitions. The volume of sample injected for analysis was 1 µL for positive ESI and 2 µL for negative ESI analysis.

###  Data analysis

MS-DIAL v5.3 software was used to process UHPLC-DIA-MS/MS data for positive and negative ion modes separately. Feature detection and alignment were performed for all samples (liver, gut and pancreas samples as well as phenotypic and pooled QCs). Parameters for feature detection (*mz*-t_R_ pairs) included minimum peak height 1000, MS1 tolerance 0.01 Da and MS2 tolerance 0.025 Da; alignment was performed with a retention time tolerance of 0.1 min and an MS1 tolerance of 0.015 Da.

For each ion mode, datasets were filtered together using the pooled QC samples across each batch to include features present in at least 50% of all pooled QCs (*n* = 15) with RSD < 20%. Precision of the analytical data was visually inspected based on QC samples; Principal Components Analysis (PCA) was performed with MetaboAnalyst v6 software. Following initial quality assurance, data acquired from each tissue were subsequently treated separately for processing and analysis. For each tissue, data were first filtered to keep only those features present in at least 50% of the phenotypic QCs (*n* = 5) with RSD < 20% to eliminate idiosyncratic behaviour as well as features specific to an alternative tissue. Next, data were filtered to include only features with > 75% presence in one of the two groups (control (*n* = 8) or ethanol treated (*n* = 8).

Tissue specific filtered datasets were analysed using MetaboAnalyst v6 software. Volcano plots of ethanol treated vs. control samples combining t-test and Log2 fold change were used to analyse the data (settings included fold change threshold 2; p-value < 0.01; group variance unequal).

All significant features were considered for metabolite annotation; precursor and product ion scanning spectra with data-independent and data-dependent acquisition methods were used to support this. MS/MS spectra were analysed using LabSolutions Insight v4.2 software (Shimadzu Corporation, Kyoto, Japan) by comparing measured data to an in-house library as well as external databases (LIPID MAPS, Metlin, mzCloud, MassBank and the HMDB). The in-house MS/MS library consisted of metabolites and lipids commonly detected with this analytical method. Library MS/MS spectra were acquired from authentic reference material when available. Complementary fragment information from each ion mode was used to provide a greater level of reporting confidence of identification: for example, the fatty acid composition of lipids could be determined from the negative ESI DDA-MS/MS data.

For structural characterisation of the significantly dysregulated lipids, OAD-MS/MS data were analysed in MS-DIAL which contains the OAD database for spectral interpretation of OAD-MS/MS data (Uchino et al., 2022) and LabSolutions Insight v4.2 software. OAD-MS/MS spectra were reviewed for chromatographic peaks found to be statistically significant in the untargeted UHPLC-MS/MS analysis. Lipid identification was performed by confirmation of CID and OAD fragments in the spectra. Lipid classifications, structural descriptions, and shorthand notations used in this study followed the definitions of LIPID MAPS. The description of C = C positions followed both delta-description and n-description and were deduced from the identification of OAD induced neutral losses previously described (Uchino et al., 2022).

## Results

### Differentiation of ethanol treated animal tissues

**Fig. 1:**
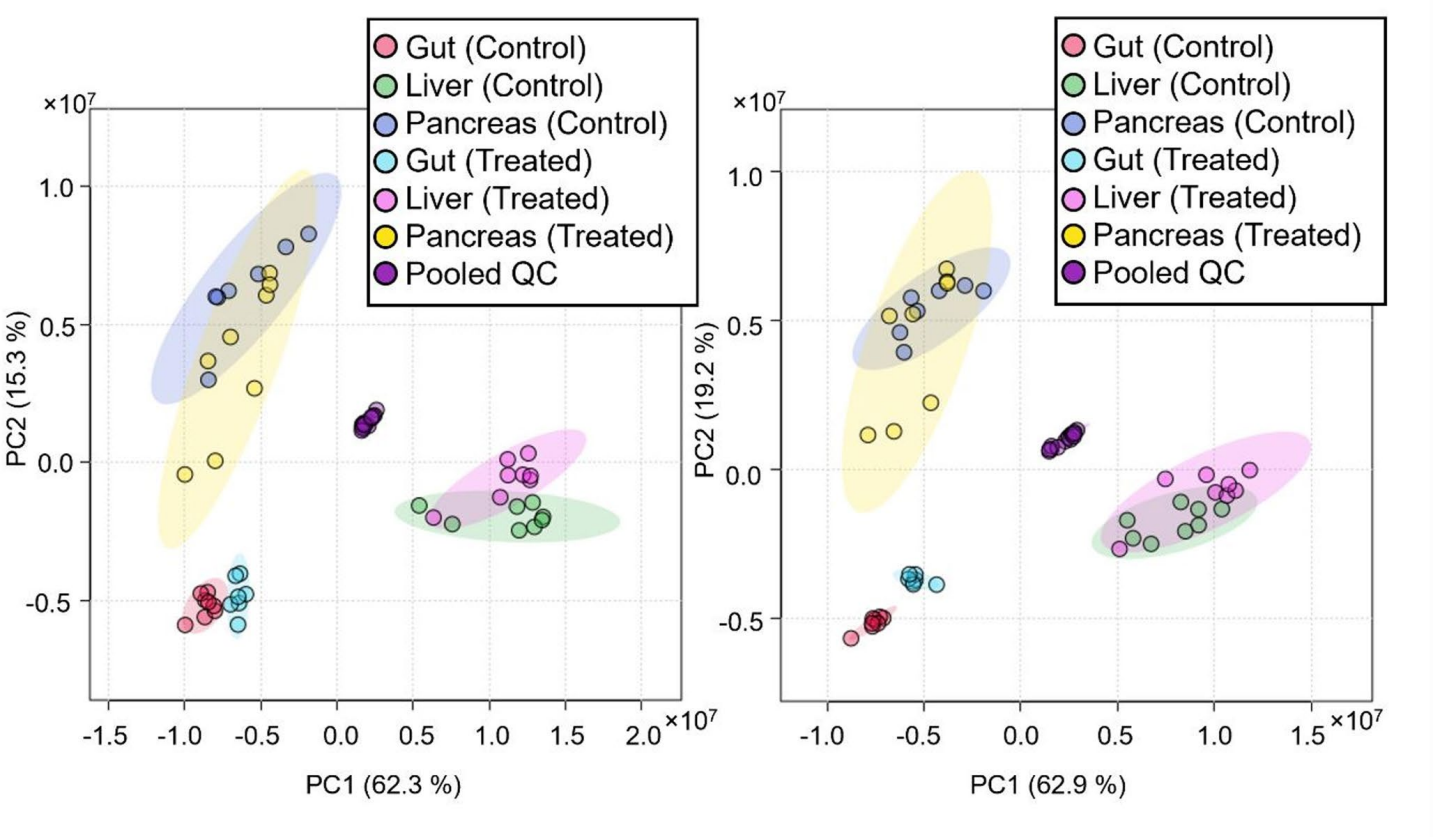
PCA score plots for features detected in positive (left) or negative ESI (right) with QC presence > 50% and RSD < 20%. MetaboAnalyst v6 software was used to generate PCA; different colours are used to highlight the control and ethanol-treated groups for each tissue (see insets to Figure)

The acquired data were analysed with the aim of discovering potential metabolic differences in mouse gut, liver and pancreas tissue extracts following acute exposure to ethanol in comparison to those of their respective controls. Following analysis by UHPLC-DIA-MS/MS data were processed to align and filter features as described in the methods. Subsequent multivariate statistical analysis (MVA) by PCA revealed two outlying samples; one gut and one liver sample from different animals, which were subsequently excluded from further analysis resulting in the PCA model shown in Fig. 1. These models, for both positive (left) and negative ESI (right) described more than 70% of the data in PCs 1 and 2).

The PCA clearly highlighted the effect of ethanol treatment on the individual tissues however, the greatest variance in these data came from tissue specificity, highlighting the importance of sampling from multiple tissues to explore the global effects of ethanol toxicity. To investigate the statistically significant effects of acute ethanol exposure on these tissue metabolomes, the data from the individual tissues were subsequently treated separately as described below.

### Dysregulated metabolites and lipids

**Fig. 2:**
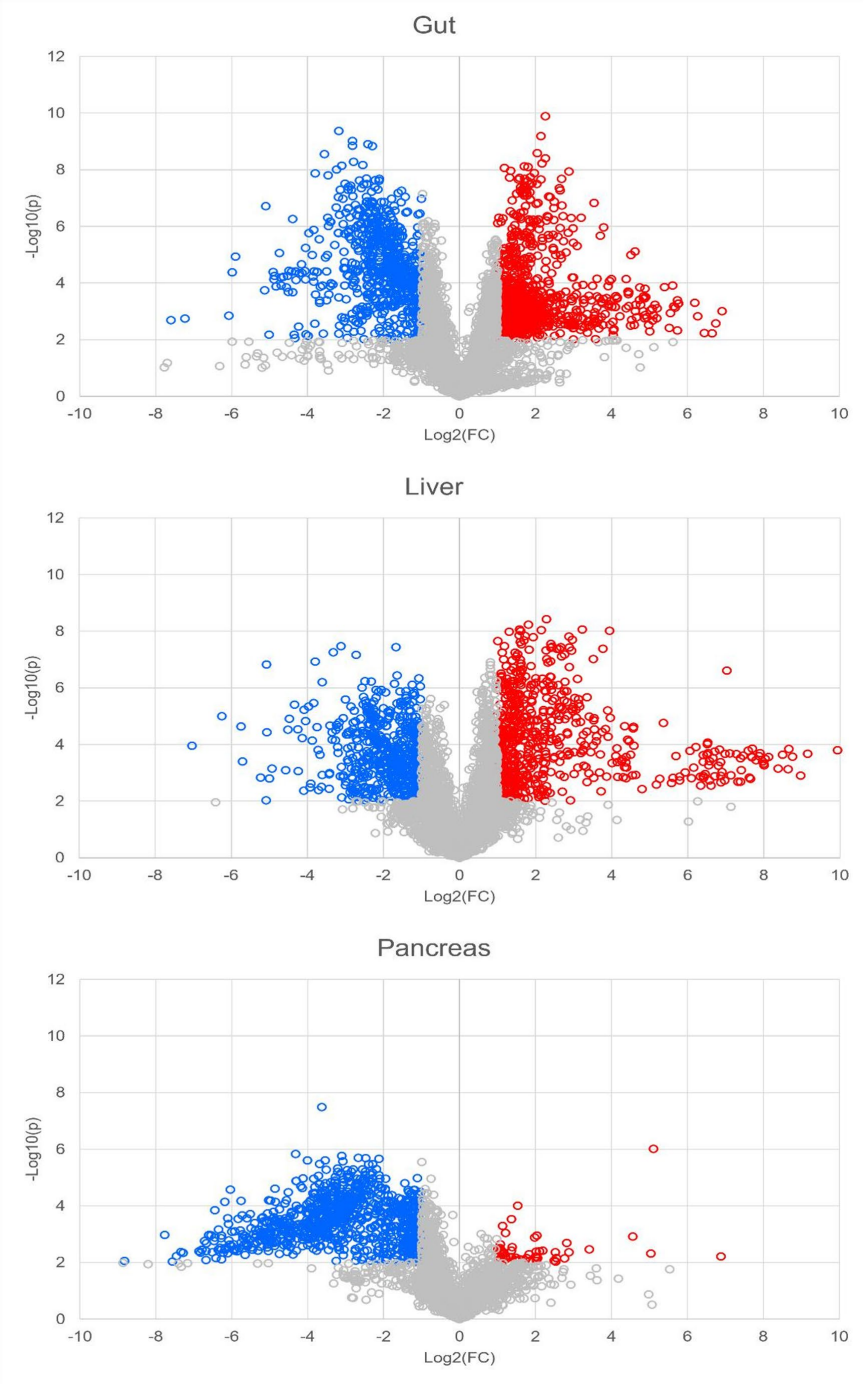
Volcano plots of features detected using UHPLC-DIA-MS/MS (positive and negative ESI combined), that were significantly increased (red) or decreased (blue) in gut (upper), liver (middle) and pancreas (lower) following ethanol administration (fold change > 2, *p* < 0.01)

**Fig. 3:**
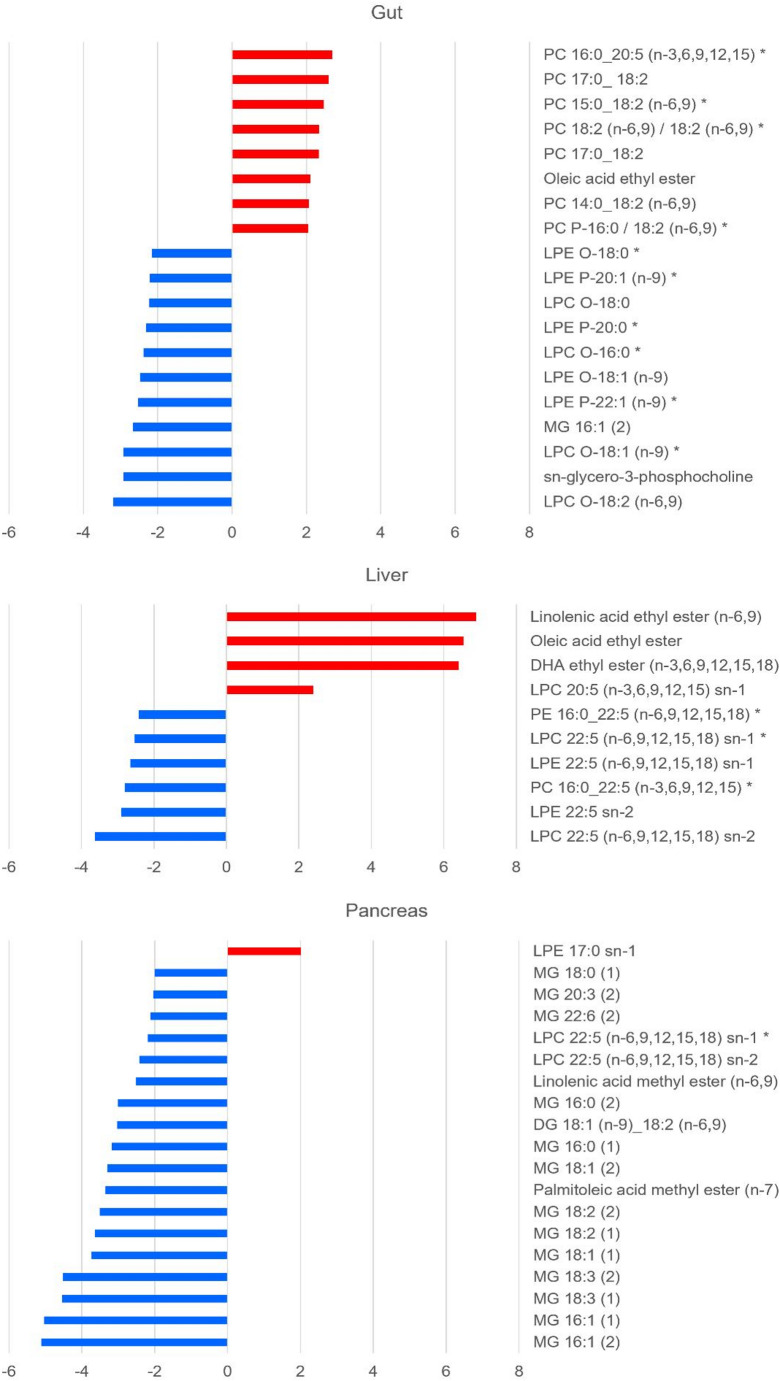
Bar-charts highlighting the most significant changes (log2 fold change > 2, *p* < 0.01) caused by acute ethanol administration in each tissue showing lipids identified to MSI levels 1 or 2 using CID and OAD-MS/MS. Those marked with (*) were significant (log2 fold change > 2, *p* < 0.01) in both positive and negative ESI

Volcano plot analysis was performed on the data for each tissue on a combined matrix of features, detected in both positive and negative ESI, following tissue specific filtering of each dataset (retaining features present in at least 50% of the tissue-specific phenotypic QCs (*n* = 5) with RSD < 20% and features with > 75% presence in one of the two groups: control or treated). Volcano plot analysis revealed significant increases in 1291, 663 and 52 features in gut, liver and pancreas respectively, while 697 features were reduced by ethanol exposure in the gut, 586 in the liver and 920 in the pancreas. The volcano plots generated for each tissue are shown in Fig. 2. Preliminary annotation of statistically significant lipids was initially performed by comparison of the DIA-MS/MS and DDA-MS/MS spectra acquired in the untargeted analysis preformed with CID-MS/MS. Spectral comparison to in-house libraries and external databases, as indicated in methods, then enabled the annotation of 101 lipids at MSI level 2. Of these, 83 lipids were unsaturated and were subsequently focussed on in the UHPLC-OAD-MS/MS data to determine where possible the location of each C = C double bond. It was possible to determine all double bonds in 61 of these 83 lipids using the OAD-MS/MS technique. Supplementary Table 1 lists the lipids that were found to be significantly dysregulated in the gut, pancreas and/or liver tissue extracts based on the volcano plot analysis. Supplementary Table 1 also provides the associated fold changes, log2 fold changes, p-values and -log10p values calculated during the volcano plot analysis for each identified feature. Significant lipids dysregulated in gut liver and/or pancreas exhibited a log2 fold change > 2 (corresponding to a fold change > 4) as a result of acute exposure to ethanol are shown in Fig. 3.

### Structural characterisation of lipid biomarkers

**Fig. 4:**
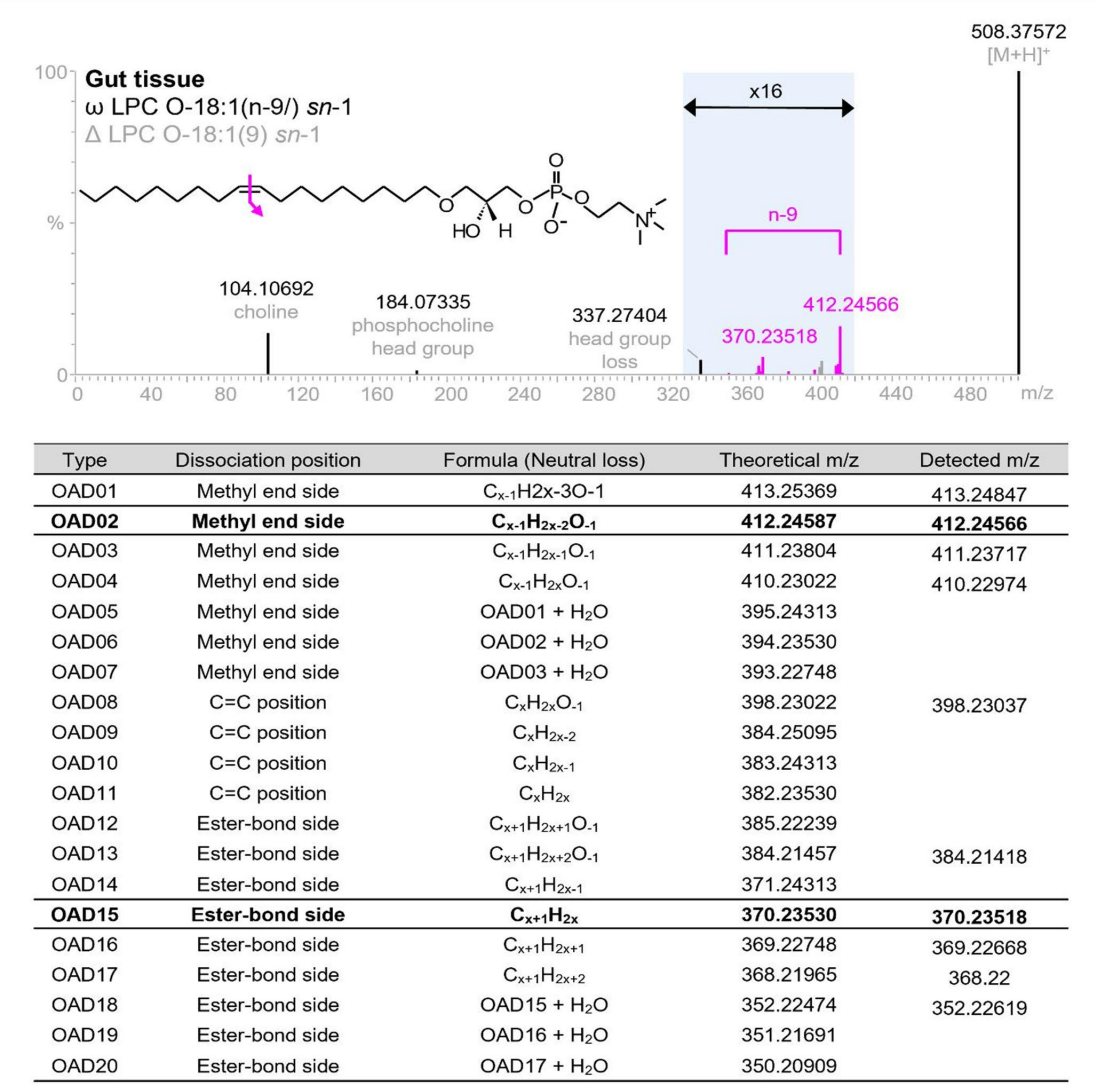
Strategy for lipid structural characterisation using OAD-MS/MS. The MS/MS spectra obtained from simultaneous OAD/CID-MS/MS analysis of the chromatographic component annotated as LPC O-18:1 sn-1 in positive ESI. Diagnostic CID fragments are highlighted in black and diagnostic OAD fragments corresponding to the double bond being in position 9 are highlighted in pink. The table presents the possible OAD fragments that could be observed for this lipid including the diagnostic pair for limit of annotation (OAD02 and OAD15) highlighted in bold. The fragments detected in the spectrum (pink) are also provided

For each of the lipids with C = C double bonds, the UHPLC-OAD-MS/MS spectral data were subsequently interrogated to determine, where possible, the position(s) of each double bond. This involved identification of OAD-MS/MS characteristic neutral loss fragments for each of the candidates as described for lipids (Uchino et al., 2022). A total of 20 possible fragments (OAD01-OAD20) can be defined for each double bond in any given lipid. The relative intensity of the various fragments can be influenced by lipid subclass, chain type and adduct, although the limit of annotation is defined as the detection of the main ‘fragment pair’ which for the instrumental set-up as described corresponds to the detection of OAD02 and OAD15. An example of OAD-MS/MS spectral interpretation that highlights this is shown in Fig. 4 for LPC O-18:1 sn-1, which was one of the lipids that was most significantly diminished by ethanol exposure in the gut tissue.

OAD-MS/MS analysis in this case enabled the identification of a C = C double bond at the 9th position. The double bond positions determined using OAD-MS/MS are denoted where applicable in the lipid names of Supplementary Table 1.

**Fig. 5:**
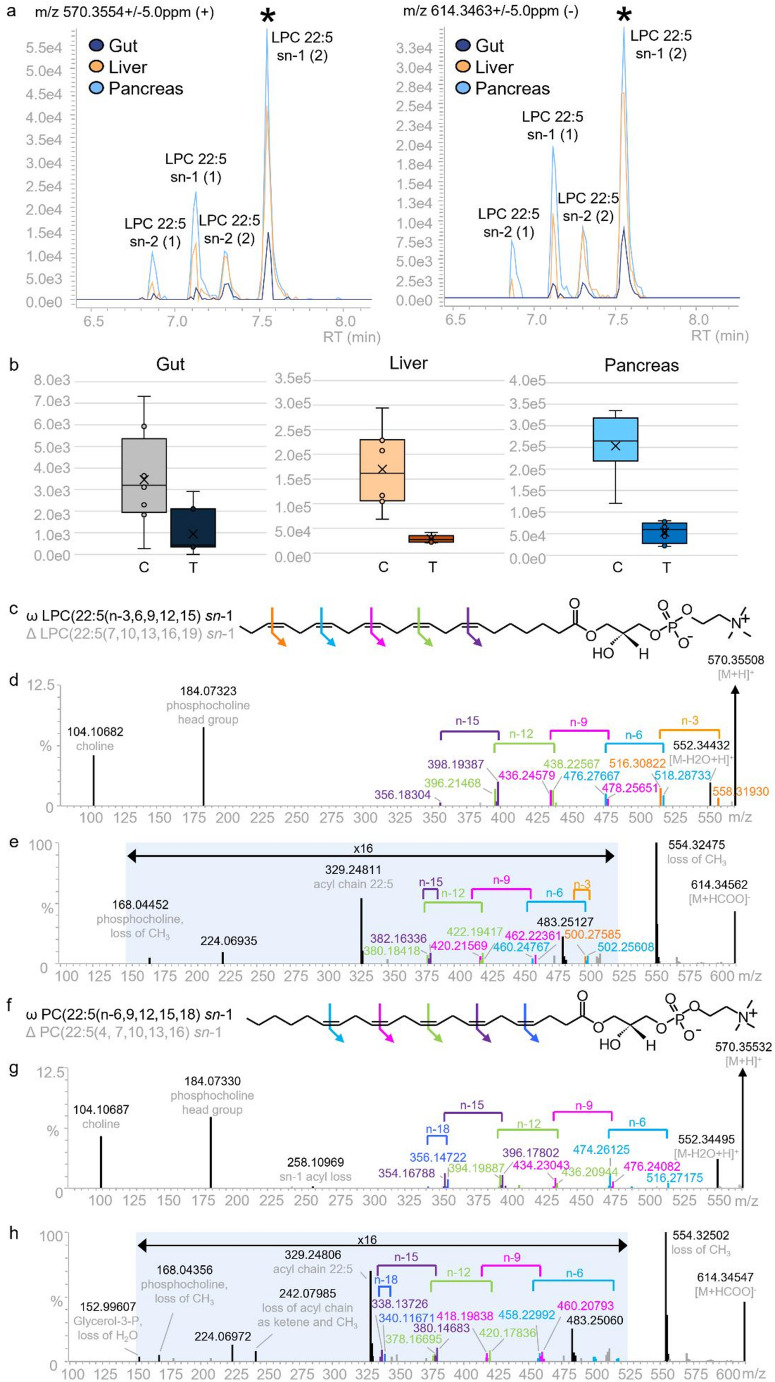
Identification of LPC 22:5 isomers. (a) MS1 extracted ion chromatograms for m/z 570.3554 [M + H] + and m/z 614 [M + HCOO-H]- in gut, liver and pancreas phenotypic tissue specific QCs analysed in the initial study; (b) Box and whisker plots of peak areas corresponding to the chromatographic peak found to be significantly diminished by ethanol exposure (LPC 22:5 sn-1 (2), C- control, T- treated with ethanol; (c) Molecular structure of LPC 22:5 (n-3,6,9,12,15) sn-1 with omega and delta nomenclature; (d) Positive ESI and (e) Negative ESI MS/MS spectra for the feature identified as LPC 22:5 sn-1 (1) in the pancreas tissue phenotypic QC. Simultaneous OAD/CID analysis specifically identifies this as LPC 22:5 (n-3,6,9,12,15) sn-1. f. Molecular structure of LPC 22:5 (n-6,9,12,15,18) sn-1 with omega and delta nomenclature; g. positive ESI and h. Negative ESI MS/MS spectra for feature identified as LPC 22:5 sn-1 (2) in the pancreatic tissue phenotypic QC. Simultaneous OAD/CID analysis specifically identified this as LPC 22:5 (n-6,9,12,15,18) sn-1

In the analysis of different tissue extracts, OAD-MS/MS enabled the structural characterisation of specific lipid isomers, indicating the exact structure affected by ethanol toxicity. An example that could be given here is that of LPC 22:5 sn-1 which was identified as being significantly reduced in relative amounts by acute exposure to ethanol in both positive ESI and negative ESI modes of analysis of liver and pancreas tissues in the initial untargeted metabolomics experiment. When inspecting the OAD-MS/MS data, this could be more specifically identified as LPC 22:5 (n-6,9,12,15,18) sn-1. As shown in Fig. 5, LPC 22:5 (n-6,9,12,15,18) sn-1 was not the only isoform of LPC 22:5 that was detected in these data. Four retention time (t_R_) separated chromatographic peaks were detected and annotated as LPC 22:5. It was possible to distinguish the sn-1 and sn-2 isoforms as two pairs using the t_R_ and ion intensities of CID fragments; the fragment at *m/z* 104 is a characterised fragment of the sn-1 isoform known to exceed a 30 fold difference in intensity relative to the same ion in the sn-2 isoform (Han & Gross, 1996). However, it was not possible to distinguish the pairs using CID-MS/MS alone. OAD-MS/MS revealed that the first set of LPC 22:5 isoforms, that were not statistically significant in this study, corresponded to LPC 22:5 (n-3,6,9, 12,15).

### Lipid distribution in gut, liver and pancreas

The analysis of gut tissue revealed the greatest number of statistically significant alterations with acute ethanol exposure, though the magnitude of log2 fold changes was generally lower than in the liver and pancreas. Notable increases in the gut tissue extracts included those of omega-6-containing phosphatidylcholines (PC 15:0_18:2(n-6,9), PC 18:2 (n-6,9)/18:2(n-6,9), PC 14:0_18:2 (n-6,9) and PC P-16:0_18:2 (n-6,9), all with log2 fold change > 2) as well as oleic acid ethyl ester (log2 fold change 2.6349). Linoleic acid (18:2 omega-6) is an essential fatty acid that exhibits a structural function in cell membranes as well as serving as a precursor to prostaglandins in inflammatory responses (Whelan & Fritsche, 2013) and linoleic acid metabolism has been previously associated with ethanol toxicity in mice (Sakallioglu et al., 2023). Significant decreases in lyso-phospholipids, particularly alkyl linked and alkenyl linked forms that are saturated or exhibit one double bond in the 9th position, were particularly characteristic of the gut tissue response to ethanol exposure (LPC O-18:2 (n-6,9), LPC O-18:1(n-9), LPE P-22:1 (n-9), LPE O-18:1(n-9) and LPE P-20:1(n-9), all with Log2 fold change <-2). These are examples of ether lipids which are involved in the regulation of cellular signalling; their quantities have been documented to be altered in various pathologies (Papin et al., 2023). Since it remains unknown whether alkyl- and alkenyl-ether lipids play differential biological roles (Papin et al., 2023), and their specific involvement in the response to acute ethanol administration has not been previously described, the discovery of these n-9 isoforms enabled by OAD-MS/MS presents a novel finding in gut tissue response.

The increase in fatty acid ethyl esters observed in the liver represented the effects with greatest magnitude in response to ethanol administration. This is consistent with the liver sustaining the greatest damage of all tissues from acute alcohol consumption because alcohol is primarily detoxified in the liver (Sakallioglu et al., 2023). Linoleic acid ethyl ester (n-6,9), oleic acid ethyl ester and docosahexaenoic acid ethyl ester (n-3,6,9,12,15,18) exhibited log2 fold changes of 6.900, 6.574 and 6.414 respectively. These specific ethyl esters were previously reported in ethanol treated rodents (Loftus et al., 2011). Although the oleic acid ethyl ester did not show definitive evidence of double bond position in the OAD-MS/MS analysis, its identification was confirmed by comparison to an authentic standard providing an MSI level 1 identification. On the other hand, OAD-MS/MS revealed the double bond positions in linoleic acid ethyl ester and docosahexaenoic acid ethyl ester which enabled an enhanced level of annotation without the availability of authentic reference material. Phospholipid responses in the liver concerned largely unsaturated lipids in which the C = C double bond positions could be determined. LPC 20:5 (n-3,6,9,12,15) sn-1 increased with log2 fold change > 2, while LPC 22:5(n-6,9,12,15,18) sn-2, LPE 22:5 sn-2, PC 16:0_22:5 (n-3,6,9,12,15), LPE 22:5(n-6,9,12,15,18) sn-1, LPC 22:5(n-6,9,12,15,18) sn-1 and PE 16:0_22:5 (n-6,9,12,15,18), were reduced with log2 fold change <-2.

In extracts of pancreatic tissue, the most significant features were decreases in monoacylglycerols with log2 fold decreases between 2 and 6. Significant reductions were observed in two chromatographically resolved isoforms of MG 16:0, 16:1, 18:1, 18:2 and 18:3. Palmitoleic acid methyl ester (n-7) and linoleic acid methyl ester were decreased (log2 fold change − 3.358 and − 2.509 respectively). In terms of phospholipids, (LPC 22:5 (n-6,9,12,15,18) sn-1 and sn-2 were the most significant. This lipid contains docosapentaenoic acid (22:5) which has previously been associated with alcohol consumption before, being reduced in myocardial tissue (Cao et al., 2021) and in the hepatic vein after alcohol intervention, indicating that circulating LPCs and specific fatty acid chains are key in the metabolism of acute alcohol intake. (Israelsen et al., 2021).

## Discussion

Lipids play pivotal roles in living organisms from their biophysical functions in cell membranes and energy storage through to their biochemical functions as hormones, receptors and signalling molecules (Conroy et al. 2024). The disruption of lipid homeostasis is associated with many diseases and has become the focus of numerous metabolomic and lipidomic studies (Ding and Rexrode 2020; Hornburg et al. 2023; Lauber et al. 2022; Pan et al. 2021). Due to the complexity and diversity of lipids that are already known, in addition to lipids yet to be discovered or identified in biological systems, the annotation of potential lipid biomarkers can be challenging and limited. Confident annotation, leading to unequivocal lipid identification, is crucial to advance our understanding of lipid biology and the roles that different lipids play in the balance between health and disease (Fernández Requena et al. 2024). Confirmation of identity using authentic standards clearly provides the highest reporting confidence for unequivocally identifying significant features in untargeted metabolomics studies, however, this is not always possible due to issues such as cost, lack of availability or poor stability of standards. Various tools are available to the lipidomics community to improve the accuracy of lipid identification (Fernández Requena et al. 2024; Goracci et al. 2017; Koelmel et al. 2020; Lange et al. 2021; Ni, Angelidou, Hoffmann, et al. 2017; Ni, Angelidou, Lange, et al. 2017), though these are typically limited by the level of fragmentation provided in the LC-CID-MS/MS data. To obtain the additional evidence required to structurally characterise lipids approaches such as ion mobility (Jeanne Dit Fouque et al. 2019; Lerner et al. 2023; Vasilopoulou et al. 2020) or alternative fragmentation methods may be of benefit. Here, the use of OAD-MS/MS as a means of enhancing of lipid annotation in discovery based untargeted metabolomics is demonstrated. While there are other alternative fragmentation methods available that are electron (Baba, Campbell, Blanc, et al. 2016; Baba, Campbell, Le Blanc, et al. 2016), photon (Ryan et al. 2017; Williams et al. 2017) or ozone-based (Barrientos et al. 2017; Marshall et al. 2019; Thomas et al. 2008), OAD provided, as shown here, an efficient means of fragmentation, especially for singly charged positive and negative ion precursors, simplifying the process of structure elucidation by generating spectra that are relatively easy to interpret.

In the mouse model of acute ethanol exposure investigated here, extracts from gut, liver and pancreatic tissue derived from ethanol-dosed and control mice were analysed by untargeted UHPLC-MS/MS analysis and further UHPLC-OAD-MS/MS in combination with DDA-MS/MS to provide structural characterisation of lipid biomarkers. Each tissue had its own distinct metabotype, whether obtained from control or alcohol-dosed mice. Ethanol exposure resulted in system - wide toxicity with divers direct and indirect effects that are responsible for damage to many organs (Birková et al. 2021). Different metabolic pathways are affected by ethanol toxicity in different tissues and several untargeted metabolomics studies have demonstrated the effects of ethanol on different tissues, particularly liver (Koelmel et al., 2021; Singh 2024; Zhao et al., 2022). Previously we have investigated the response of the liver to ethanol toxicity in rats and mice, finding major alterations in glycerol lipids, fatty acyls, fatty acid ethyl esters, and phosphatidylethanol homologues (Loftus et al., 2011). To explore the effects of ethanol toxicity beyond the liver, we investigated the metabolic alterations ethanol toxicity induced in plasma and urine (Gika et al., 2012) as well as the effects to the brains of mice administered acute and chronic levels of ethanol (Deda et al., 2020). In brain tissue, the response was distinct, centring on amino acid metabolism as well as marked alterations in nucleotides, nucleosides and related metabolites.

In the present study, there was a large impact in the metabolic phenotype of ethanol exposed animals with numerous lipids found to be significantly altered in all three tissues. However, these alterations in lipid metabotypes were tissue specific with only LPC 20:4 (n-6,9,12,15) sn-2 highlighted as being statistically significantly changed in all three tissues. Even in the case of this LPC we found that firstly, it was not the most significant result for any tissue, with Log2 FC < 2, and whilst this lipid was relatively increased in gut, the opposite was true for pancreas and liver.

The complex picture of lipid (and other) dysregulated metabolites across the 3 tissues studied here, plus those previously reported for these mice for brain and blood plasma represents a complex picture of the response of the various organs to acute ethanol exposure. Clearly further analysis of these data is required to be able to integrate the various effects seen here to better understand the biochemistry underlying these issue specific changes.

## Conclusions

Reversed-phase UHPLC-DIA-MS/MS analysis revealed significant alterations in lipid profiles from the gut, liver and pancreatic tissue of mice undergoing acute dietary exposure to ethanol compared to control animals. Each of the different tissues, irrespective of whether they came from control or ethanol-treated mice, provided distinct and specific metabolic phenotypes. Many of the metabolites affected by exposure to ethanol were found to be lipids and due to the diversity and complexity of the profiles obtained their unequivocal identification, rather than mere annotation, represented a significant challenge. UHPLC-DIA-MS/MS analysis of all samples and DDA-MS/MS analysis of phenotypic tissue QCs enabled the annotation of 101 lipids that were significantly dysregulated in the gut, liver and/or pancreas by ethanol administration. A total of 83 of these lipids corresponded to unsaturated lipids and were further analysed using UHPLC-OAD-MS/MS. Using this technique, the sites of the C = C double bonds could be located in 61 of the unsaturated lipids. These unsaturated lipids included isomers that were indistinguishable by CID-MS/MS. The ability to identify multiple lipid isoforms increases the scope for their annotation and identification in complex biological samples and thereby improves the confidence with which reporting to MSI level 2 identification can be made.

## Electronic supplementary material

Below is the link to the electronic supplementary material.

Supplementary Material 1

Supplementary Material 2

## Data Availability

All relevant metabolomics data are provided in the Supplementary Information
