# Supplementary material for: Oxygen attachment dissociation (OAD) MS/MS in the identification of positional isomers of dysregulated lipids detected in an ethanol exposure metabolomics study in mice
Source: Metabolomics. 2025 Jul 1;21(4):95. doi: 10.1007/s11306-025-02282-8 (PMC12213981; doi:10.1007/s11306-025-02282-8)
Supplement: Supplementary file 2 — Supplementary Material 2 [file 11306_2025_2282_MOESM2_ESM.docx]

Supplementary Information for article:

Oxygen Attachment Dissociation (OAD) MS/MS in the identification of positional isomers of dysregulated lipids detected in an ethanol exposure metabolomics study in mice

Journal: Metabolomics

Authors:

Emily G Armitage^1^; Alan Barnes^1^; Olga Deda^2,3^; Christina Virgiliou^3,4^; Neil J Loftus^1^; Helen Gika^2,3^; Ian D Wilson^5^

Affiliations:

1 Shimadzu Corporation, Manchester, M17 1GP, UK

2 Department of Medicine, Aristotle University of Thessaloniki, 54124 Thessaloniki, Greece

3 BiOMIC_AUTh, Centre for Interdisciplinary Research and Innovation (CIRI-AUTH), Balkan Centre, 57001 Thessaloniki, Greece

4 Department of Chemical Engineering, Aristotle University of Thessaloniki, 54124 Thessaloniki, Greece

5 Computational & Systems Medicine, Department of Metabolism, Digestion and Reproduction, Imperial College, Burlington Danes Building, Du Cane Road, London, W12 0NN, UK

Corresponding author: Ian D Wilson. E-mail: ianwilsonprof@gmail.com

**Supplementary table** 1

Annotated significant results from LC-MS/MS untargeted metabolic profiling of mouse gut liver and pancreas following chronic ethanol exposure. Annotations represent those determined by spectral comparison of DIA-MS/MS and DDA-MS/MS spectra obtained through metabolic profiling using the standard method with Collision induced dissociation to in-house libraries and external databases (LIPID MAPS, Metlin, mzcloud, MassBank and HMDB). The level of confidence in identification as determined by the Metabolomics Standards Initiative (MSI level) is presented. The identification of many lipids was enhanced by the determination of C=C bond positions in the analysis with OAD-MS/MS. The double bond positions are given in the annotation where applicable. The tissue column shows which tissues the significant result was found and the subsequent columns FC (fold change), log2(FC), p-value and -log10(p) are the values generated by volcano plot analysis of each tissue using MetaboAnalyst v6 software. The direction of change is denoted by the log2(FC); positive values indicate an increase in samples from ethanol treated mice while negative values indicate a decrease compared to controls.

| Lipid name | Formula | Detected m/z | Detected RT | Theoretical m/z | Ion | Tissue | FC | log2(FC) | p-value | -log10(p) | Control mean | Control SD | Treated mean | Treated SD |
| --- | --- | --- | --- | --- | --- | --- | --- | --- | --- | --- | --- | --- | --- | --- |
| DG 18:1(n-9)_18:2(n-6,9) | C39H70O5 | 641.5114 | 29.271 | 641.51155 | [M+Na]+ | Gut | 0.253 | -1.984 | 1.9E-04 | 3.712 | 62922 | 22871 | 15903 | 8351 |
| DG 18:1(n-9)_18:2(n-6,9) | C39H70O5 | 641.5114 | 29.271 | 641.51155 | [M+Na]+ | Pancreas | 0.122 | -3.032 | 1.2E-04 | 3.936 | 330550 | 148000 | 40409 | 32427 |
| Docosahexaenoic acid ethyl ester (n-3,6,9,12,15,18) | C24H36O2 | 357.2783 | 14.488 | 357.27881 | [M+H]+ | Liver | 85.275 | 6.414 | 1.5E-03 | 2.819 | 2741 | 2955 | 233695 | 166286 |
| Linoleic acid (n-6,9) | C18H32O2 | 281.2474 | 11.044 | 281.24751 | [M+H]+ | Gut | 0.472 | -1.084 | 1.4E-04 | 3.861 | 694978 | 156009 | 327768 | 100334 |
| Linoleic acid ethyl ester (n-6,9) | C20H36O2 | 309.2783 | 15.768 | 309.27881 | [M+H]+ | Liver | 119.410 | 6.900 | 9.4E-04 | 3.026 | 1041 | 866 | 124247 | 83540 |
| Linolenic acid methyl ester (n-6,9) | C19H32O2 | 293.2472 | 12.872 | 293.24751 | [M+H]+ | Pancreas | 0.176 | -2.509 | 3.9E-03 | 2.410 | 89792 | 59184 | 15775 | 9634 |
| LPC 17:0 sn-1 | C25H52NO7P | 510.3557 | 8.267 | 510.35542 | [M+H]+ | Gut | 2.105 | 1.074 | 1.0E-04 | 3.993 | 21256 | 7878 | 44738 | 8654 |
| LPC 17:0 sn-1 | C25H52NO7P | 554.3454 | 8.259 | 554.34634 | [M+HCOO-H]- | Gut | 2.302 | 1.203 | 6.0E-06 | 5.225 | 20007 | 6018 | 46052 | 7782 |
| LPC 17:0 sn-1 | C25H52NO7P | 554.3454 | 8.259 | 554.34634 | [M+HCOO-H]- | Pancreas | 2.256 | 1.174 | 4.8E-03 | 2.320 | 12535 | 2919 | 28283 | 11911 |
| LPC 18:0 sn-2 | C26H54NO7P | 524.3707 | 8.814 | 524.37107 | [M+H]+ | Liver | 2.012 | 1.009 | 2.2E-08 | 7.656 | 171737 | 28209 | 345604 | 33552 |
| LPC 18:1(n-9) sn-1 | C26H52NO7P | 566.3470 | 7.809 | 566.34634 | [M+HCOO-H]- | Gut | 2.145 | 1.101 | 2.0E-04 | 3.697 | 190238 | 56304 | 408074 | 104937 |
| LPC 18:1(n-9) sn-2 | C26H52NO7P | 522.3560 | 7.493 | 522.35542 | [M+H]+ | Gut | 2.639 | 1.400 | 7.9E-06 | 5.102 | 25988 | 5983 | 68582 | 15759 |
| LPC 18:1(n-9) sn-2 | C26H52NO7P | 566.3466 | 7.486 | 566.34634 | [M+HCOO-H]- | Gut | 2.814 | 1.493 | 8.2E-06 | 5.085 | 27845 | 6496 | 78352 | 19019 |
| LPC 18:2(n-6,9) sn-1 | C26H50NO7P | 520.3413 | 6.775 | 520.33977 | [M+H]+ | Gut | 2.128 | 1.090 | 2.1E-03 | 2.685 | 74461 | 32902 | 158451 | 51165 |
| LPC 18:2(n-6,9) sn-1 | C26H50NO7P | 564.3399 | 6.771 | 564.33069 | [M+HCOO-H]- | Gut | 2.340 | 1.226 | 1.7E-03 | 2.769 | 64181 | 28468 | 150173 | 53971 |
| LPC 18:2(n-6,9) sn-1 | C26H50NO7P | 542.3214 | 6.771 | 542.32171 | [M+Na]+ | Gut | 2.206 | 1.141 | 1.6E-03 | 2.802 | 3767 | 1817 | 8309 | 2588 |
| LPC 18:2(n-6,9) sn-1 | C26H50NO7P | 542.3214 | 6.771 | 542.32171 | [M+Na]+ | Liver | 2.013 | 1.010 | 1.3E-04 | 3.900 | 156266 | 25418 | 314632 | 81675 |
| LPC 18:2(n-6,9) sn-2 | C26H50NO7P | 520.3411 | 6.495 | 520.33977 | [M+H]+ | Gut | 3.274 | 1.711 | 3.3E-05 | 4.487 | 11455 | 3168 | 37505 | 11466 |
| LPC 18:2(n-6,9) sn-2 | C26H50NO7P | 564.3316 | 6.490 | 564.33069 | [M+HCOO-H]- | Gut | 4.058 | 2.021 | 1.3E-04 | 3.900 | 9265 | 3404 | 37598 | 14526 |
| LPC 18:3(n-6,9,12) sn-1 | C26H48NO7P | 518.3234 | 6.111 | 518.32412 | [M+H]+ | Pancreas | 0.491 | -1.025 | 7.9E-04 | 3.104 | 54585 | 12354 | 26822 | 12313 |
| LPC 20:3(n-6,9,12) sn-1 | C28H52NO7P | 546.3553 | 7.362 | 546.35542 | [M+H]+ | Liver | 2.029 | 1.021 | 4.4E-04 | 3.355 | 149366 | 59027 | 303054 | 74756 |
| LPC 20:3(n-6,9,12) sn-2 | C28H52NO7P | 546.3555 | 7.085 | 546.35542 | [M+H]+ | Gut | 2.769 | 1.469 | 2.1E-05 | 4.684 | 3201 | 990 | 8863 | 2244 |
| LPC 20:3(n-6,9,12) sn-2 | C28H52NO7P | 590.3459 | 7.079 | 590.34634 | [M+HCOO-H]- | Gut | 2.712 | 1.439 | 4.5E-05 | 4.343 | 3133 | 725 | 8496 | 2424 |
| LPC 20:4(n-6,9,12,15) sn-1 | C28H50NO7P | 588.3316 | 6.769 | 588.33069 | [M+HCOO-H]- | Pancreas | 0.497 | -1.009 | 5.7E-04 | 3.245 | 2559317 | 467331 | 1271881 | 611336 |
| LPC 20:4(n-6,9,12,15) sn-2 | C28H50NO7P | 544.3405 | 6.531 | 544.33977 | [M+H]+ | Gut | 2.118 | 1.083 | 4.4E-04 | 3.353 | 13011 | 3096 | 27562 | 8220 |
| LPC 20:4(n-6,9,12,15) sn-2 | C28H50NO7P | 544.3405 | 6.531 | 544.33977 | [M+H]+ | Liver | 0.310 | -1.691 | 1.3E-04 | 3.874 | 1062693 | 384371 | 329161 | 105858 |
| LPC 20:4(n-6,9,12,15) sn-2 | C28H50NO7P | 544.3405 | 6.531 | 544.33977 | [M+H]+ | Pancreas | 0.335 | -1.576 | 9.3E-05 | 4.031 | 1671798 | 410738 | 560704 | 364410 |
| LPC 20:4(n-6,9,12,15) sn-2 | C28H50NO7P | 588.3313 | 6.525 | 588.33069 | [M+HCOO-H]- | Gut | 2.221 | 1.151 | 3.9E-04 | 3.409 | 12486 | 3305 | 27733 | 8434 |
| LPC 20:4(n-6,9,12,15) sn-2 | C28H50NO7P | 588.3313 | 6.525 | 588.33069 | [M+HCOO-H]- | Liver | 0.301 | -1.730 | 1.2E-04 | 3.923 | 948092 | 341210 | 285832 | 100702 |
| LPC 20:4(n-6,9,12,15) sn-2 | C28H50NO7P | 588.3313 | 6.525 | 588.33069 | [M+HCOO-H]- | Pancreas | 0.357 | -1.486 | 1.3E-04 | 3.880 | 1478125 | 345125 | 527538 | 341600 |
| LPC 20:5(n-3,6,9,12,15) sn-1 | C28H48NO7P | 542.3235 | 6.007 | 542.32412 | [M+H]+ | Liver | 5.283 | 2.401 | 3.9E-08 | 7.413 | 9162 | 3908 | 48405 | 9574 |
| LPC 22:4(6,9,12,15) sn-1 | C30H54NO7P | 572.3708 | 8.014 | 572.37107 | [M+H]+ | Pancreas | 0.481 | -1.055 | 8.3E-04 | 3.079 | 142111 | 30133 | 68381 | 35244 |
| LPC 22:4(6,9,12,15) sn-2 | C30H54NO7P | 572.3709 | 7.738 | 572.37107 | [M+H]+ | Liver | 0.286 | -1.805 | 2.1E-03 | 2.669 | 10495 | 5374 | 3003 | 1731 |
| LPC 22:4(6,9,12,15) sn-2 | C30H54NO7P | 572.3709 | 7.738 | 572.37107 | [M+H]+ | Pancreas | 0.359 | -1.476 | 4.7E-04 | 3.327 | 51457 | 16390 | 18497 | 11007 |
| LPC 22:4(6,9,12,15) sn-2 | C30H54NO7P | 616.3611 | 7.728 | 616.36199 | [M+HCOO]- | Pancreas | 0.364 | -1.459 | 3.5E-04 | 3.457 | 42988 | 13198 | 15642 | 8720 |
| LPC 22:5(n-6,9,12,15,18) sn-1 | C30H52NO7P | 570.3549 | 7.556 | 570.35542 | [M+H]+ | Liver | 0.173 | -2.529 | 2.1E-04 | 3.684 | 171781 | 80485 | 29760 | 8074 |
| LPC 22:5(n-6,9,12,15,18) sn-1 | C30H52NO7P | 570.3549 | 7.556 | 570.35542 | [M+H]+ | Pancreas | 0.220 | -2.185 | 5.0E-06 | 5.298 | 255458 | 72084 | 56190 | 23351 |
| LPC 22:5(n-6,9,12,15,18) sn-1 | C30H52NO7P | 614.3453 | 7.547 | 614.34634 | [M+HCOO-H]- | Liver | 0.162 | -2.630 | 4.6E-04 | 3.340 | 131307 | 68307 | 21214 | 5151 |
| LPC 22:5(n-6,9,12,15,18) sn-2 | C30H52NO7P | 614.3455 | 7.307 | 614.34634 | [M+HCOO-H]- | Liver | 0.081 | -3.633 | 3.2E-03 | 2.500 | 43966 | 32109 | 3545 | 1620 |
| LPC 22:5(n-6,9,12,15,18) sn-1 | C30H52NO7P | 614.3453 | 7.547 | 614.34634 | [M+HCOO-H]- | Pancreas | 0.230 | -2.119 | 8.2E-06 | 5.087 | 204235 | 59864 | 47004 | 18406 |
| LPC 22:5(n-6,9,12,15,18) sn-2 | C30H52NO7P | 614.3455 | 7.307 | 614.34634 | [M+HCOO-H]- | Pancreas | 0.186 | -2.426 | 2.4E-04 | 3.613 | 55402 | 25103 | 10312 | 4929 |
| LPC 22:6(n-3,6,9,12,15,18) sn-2 | C30H50NO7P | 568.3394 | 6.492 | 568.33977 | [M+H]+ | Liver | 0.496 | -1.011 | 9.8E-03 | 2.009 | 615773 | 256777 | 305595 | 142464 |
| LPC O-16:0 | C24H52NO6P | 482.3601 | 7.844 | 482.36050 | [M+H]+ | Gut | 0.192 | -2.383 | 3.5E-07 | 6.455 | 322218 | 61673 | 61775 | 41509 |
| LPC O-16:0 | C24H52NO6P | 526.3499 | 7.837 | 526.35143 | [M+HCOO-H]- | Gut | 0.205 | -2.284 | 1.6E-06 | 5.786 | 325775 | 70542 | 66908 | 46873 |
| LPC O-18:0 | C26H56NO6P | 510.3918 | 9.755 | 510.39180 | [M+H]+ | Gut | 0.214 | -2.224 | 4.2E-08 | 7.382 | 95955 | 13440 | 20535 | 12153 |
| LPC O-18:1(n-9) | C26H54NO6P | 508.3758 | 8.253 | 508.37615 | [M+H]+ | Gut | 0.132 | -2.925 | 4.0E-08 | 7.399 | 580969 | 105680 | 76499 | 53858 |
| LPC O-18:1(n-9) | C26H54NO6P | 552.3657 | 8.247 | 552.36708 | [M+HCOO-H]- | Gut | 0.131 | -2.927 | 2.2E-07 | 6.649 | 530533 | 114418 | 69742 | 50677 |
| LPC O-18:2(n-6,9) | C26H52NO6P | 506.3599 | 7.135 | 506.36050 | [M+H]+ | Gut | 0.109 | -3.193 | 9.1E-08 | 7.039 | 24533 | 4947 | 2682 | 2416 |
| LPC P-16:0 | C24H50NO6P | 480.3449 | 7.838 | 480.34485 | [M+H]+ | Gut | 0.367 | -1.446 | 9.9E-06 | 5.005 | 668610 | 95745 | 245422 | 138523 |
| LPC P-16:0 | C24H50NO6P | 524.3348 | 7.831 | 524.33578 | [M+HCOO-H]- | Gut | 0.392 | -1.352 | 4.0E-05 | 4.402 | 598590 | 100528 | 234567 | 131502 |
| LPC P-18:1(n-9) | C26H52NO6P | 506.3605 | 8.260 | 506.36050 | [M+H]+ | Gut | 0.354 | -1.498 | 2.1E-06 | 5.668 | 165454 | 22054 | 58561 | 29433 |
| LPC P-18:1(n-9) | C26H52NO6P | 550.3495 | 8.249 | 550.35143 | [M+HCOO-H]- | Gut | 0.369 | -1.440 | 4.6E-06 | 5.334 | 137449 | 19432 | 50662 | 25465 |
| LPE 16:0 sn-1 | C21H44NO7P | 452.2784 | 7.325 | 452.27826 | [M-H]- | Pancreas | 2.088 | 1.062 | 4.0E-03 | 2.399 | 381976 | 138217 | 797693 | 286410 |
| LPE 17:0 sn-1 | C22H46NO7P | 468.3082 | 8.206 | 468.30847 | [M+H]+ | Liver | 2.146 | 1.102 | 8.5E-05 | 4.069 | 26185 | 13107 | 56190 | 8398 |
| LPE 17:0 sn-1 | C22H46NO7P | 466.2929 | 8.196 | 466.29391 | [M-H]- | Liver | 2.144 | 1.100 | 4.4E-04 | 3.360 | 37609 | 22700 | 80638 | 13928 |
| LPE 17:0 sn-1 | C22H46NO7P | 466.2929 | 8.196 | 466.29391 | [M-H]- | Pancreas | 4.046 | 2.016 | 4.1E-03 | 2.385 | 3814 | 1480 | 15432 | 8699 |
| LPE 18:2(n-6,9) sn-1 | C23H44NO7P | 478.2932 | 6.717 | 478.29282 | [M+H]+ | Gut | 2.076 | 1.054 | 6.3E-03 | 2.201 | 25716 | 8750 | 53377 | 22260 |
| LPE 18:2(n-6,9) sn-1 | C23H44NO7P | 478.2932 | 6.717 | 478.29282 | [M+H]+ | Liver | 3.092 | 1.628 | 2.9E-08 | 7.537 | 163019 | 40331 | 503987 | 78038 |
| LPE 18:2(n-6,9) sn-1 | C23H44NO7P | 476.2791 | 6.712 | 476.27826 | [M-H]- | Gut | 2.205 | 1.141 | 8.5E-03 | 2.071 | 33920 | 10523 | 74805 | 35787 |
| LPE 18:2(n-6,9) sn-1 | C23H44NO7P | 476.2791 | 6.712 | 476.27826 | [M-H]- | Liver | 2.956 | 1.564 | 1.4E-08 | 7.856 | 218527 | 56749 | 645921 | 87053 |
| LPE 18:2(n-6,9) sn-2 | C23H44NO7P | 478.2926 | 6.441 | 478.29282 | [M+H]+ | Gut | 3.101 | 1.633 | 4.6E-04 | 3.342 | 3860 | 1109 | 11971 | 4815 |
| LPE 18:2(n-6,9) sn-2 | C23H44NO7P | 476.2780 | 6.434 | 476.27826 | [M-H]- | Gut | 3.017 | 1.593 | 4.8E-04 | 3.315 | 6096 | 1213 | 18390 | 7462 |
| LPE 20:3(n-6,9,12) sn-1 | C25H46NO7P | 504.3081 | 7.309 | 504.30847 | [M+H]+ | Liver | 2.156 | 1.109 | 9.4E-05 | 4.025 | 21950 | 11124 | 47328 | 7297 |
| LPE 20:3(n-6,9,12) sn-1 | C25H46NO7P | 502.2933 | 7.301 | 502.29391 | [M-H]- | Liver | 2.143 | 1.100 | 7.2E-05 | 4.140 | 25359 | 12467 | 54344 | 7959 |
| LPE 22:5(n-6,9,12,15,18) sn-1 | C27H46NO7P | 528.3086 | 7.505 | 528.30847 | [M+H]+ | Gut | 0.371 | -1.430 | 1.3E-04 | 3.893 | 18364 | 3601 | 6814 | 4725 |
| LPE 22:5(n-6,9,12,15,18) sn-1 | C27H46NO7P | 528.3086 | 7.505 | 528.30847 | [M+H]+ | Pancreas | 0.371 | -1.429 | 1.6E-04 | 3.802 | 453989 | 139012 | 168620 | 62809 |
| LPE 22:5(n-6,9,12,15,18) sn-1 | C27H46NO7P | 526.2935 | 7.497 | 526.29391 | [M-H]- | Gut | 0.428 | -1.224 | 1.0E-03 | 2.991 | 22161 | 3380 | 9484 | 7748 |
| LPE 22:5(n-6,9,12,15,18) sn-1 | C27H46NO7P | 526.2935 | 7.497 | 526.29391 | [M-H]- | Pancreas | 0.392 | -1.350 | 2.9E-04 | 3.534 | 558632 | 177531 | 219208 | 79445 |
| LPE 22:5(n-6,9,12,15,18) sn-2 | C27H46NO7P | 528.3085 | 7.259 | 528.30847 | [M+H]+ | Liver | 0.133 | -2.910 | 8.6E-03 | 2.066 | 24830 | 19862 | 3304 | 1794 |
| LPE 22:5(n-6,9,12,15,18) sn-2 | C27H46NO7P | 528.3085 | 7.259 | 528.30847 | [M+H]+ | Pancreas | 0.355 | -1.492 | 2.5E-03 | 2.606 | 98534 | 45539 | 35027 | 14909 |
| LPE 22:5(n-6,9,12,15,18) sn-2 | C27H46NO7P | 526.2935 | 7.252 | 526.29391 | [M-H]- | Liver | 0.159 | -2.653 | 9.8E-03 | 2.008 | 30271 | 24050 | 4814 | 1699 |
| LPE 22:5(n-6,9,12,15,18) sn-2 | C27H46NO7P | 526.2935 | 7.252 | 526.29391 | [M-H]- | Pancreas | 0.379 | -1.401 | 1.7E-03 | 2.771 | 120517 | 50182 | 45622 | 18641 |
| LPE O-18:0 | C23H50NO6P | 468.3431 | 9.691 | 468.34485 | [M+H]+ | Gut | 0.224 | -2.160 | 1.3E-06 | 5.874 | 238773 | 49267 | 53423 | 33522 |
| LPE O-18:0 | C23H50NO6P | 466.3270 | 9.683 | 466.33030 | [M-H]- | Gut | 0.236 | -2.081 | 4.1E-06 | 5.382 | 380599 | 87303 | 89937 | 55442 |
| LPE O-18:1(n-9) | C23H48NO6P | 464.3122 | 8.194 | 464.31465 | [M-H]- | Gut | 0.180 | -2.473 | 4.3E-07 | 6.366 | 484741 | 100255 | 87301 | 56079 |
| LPE O-18:1(n-9) | C23H48NO6P | 464.3122 | 8.194 | 464.31465 | [M-H]- | Pancreas | 2.811 | 1.491 | 8.9E-03 | 2.051 | 16142 | 4391 | 45376 | 24712 |
| LPE P-16:0 | C21H44NO6P | 438.2988 | 7.788 | 438.29790 | [M+H]+ | Gut | 0.327 | -1.615 | 5.0E-06 | 5.300 | 2137668 | 221851 | 698039 | 496697 |
| LPE P-16:0 | C21H44NO6P | 436.2830 | 7.781 | 436.28335 | [M-H]- | Gut | 0.370 | -1.434 | 2.6E-05 | 4.581 | 2590908 | 331715 | 958906 | 640094 |
| LPE P-16:0 | C21H44NO6P | 436.2830 | 7.781 | 436.28335 | [M-H]- | Pancreas | 2.798 | 1.484 | 6.8E-03 | 2.167 | 368770 | 96864 | 1031665 | 536060 |
| LPE P-17:0 | C22H46NO6P | 452.3137 | 8.700 | 452.31355 | [M+H]+ | Gut | 0.262 | -1.931 | 3.6E-06 | 5.438 | 113555 | 22474 | 29772 | 19528 |
| LPE P-17:0 | C22H46NO6P | 450.2980 | 8.693 | 450.29900 | [M-H]- | Gut | 0.282 | -1.824 | 2.8E-05 | 4.554 | 159163 | 38526 | 44952 | 30605 |
| LPE P-18:0 | C23H48NO6P | 466.3296 | 9.682 | 466.32920 | [M+H]+ | Gut | 0.289 | -1.793 | 1.4E-06 | 5.868 | 1719421 | 247703 | 496109 | 318027 |
| LPE P-18:0 | C23H48NO6P | 464.3144 | 9.673 | 464.31465 | [M-H]- | Gut | 0.325 | -1.623 | 3.9E-06 | 5.411 | 2240067 | 338296 | 727093 | 432085 |
| LPE P-18:1(n-9) | C23H46NO6P | 464.3140 | 8.210 | 464.31355 | [M+H]+ | Gut | 0.307 | -1.704 | 2.7E-07 | 6.563 | 2274911 | 215451 | 698158 | 402548 |
| LPE P-18:1(n-9) | C23H46NO6P | 462.2984 | 8.202 | 462.29900 | [M-H]- | Gut | 0.349 | -1.518 | 2.3E-06 | 5.631 | 2792408 | 341263 | 975033 | 533923 |
| LPE P-18:2(n-6,9) | C23H44NO6P | 462.2976 | 7.120 | 462.29790 | [M+H]+ | Gut | 0.252 | -1.987 | 1.4E-07 | 6.867 | 211871 | 26389 | 53462 | 33490 |
| LPE P-18:2(n-6,9) | C23H44NO6P | 460.2818 | 7.112 | 460.28335 | [M-H]- | Gut | 0.256 | -1.963 | 2.6E-06 | 5.581 | 263973 | 52060 | 67705 | 43028 |
| LPE P-18:2(n-6,9) | C23H44NO6P | 460.2818 | 7.112 | 460.28335 | [M-H]- | Pancreas | 2.566 | 1.360 | 6.5E-03 | 2.186 | 36869 | 8955 | 94612 | 46270 |
| LPE P-20:0 | C25H52NO6P | 494.3605 | 11.858 | 494.36050 | [M+H]+ | Gut | 0.202 | -2.310 | 1.6E-07 | 6.803 | 308410 | 55243 | 62203 | 35082 |
| LPE P-20:0 | C25H52NO6P | 492.3446 | 11.845 | 492.34595 | [M-H]- | Gut | 0.222 | -2.171 | 8.3E-07 | 6.083 | 430837 | 87841 | 95682 | 53486 |
| LPE P-20:1(n-9) | C25H50NO6P | 492.3445 | 10.013 | 492.34485 | [M+H]+ | Gut | 0.216 | -2.213 | 2.3E-08 | 7.637 | 73684 | 10486 | 15888 | 7905 |
| LPE P-20:1(n-9) | C25H50NO6P | 490.3295 | 10.004 | 490.33030 | [M-H]- | Gut | 0.235 | -2.090 | 6.4E-07 | 6.192 | 104960 | 19745 | 24658 | 14018 |
| LPE P-22:1(n-9) | C27H54NO6P | 520.3755 | 12.128 | 520.37615 | [M+H]+ | Gut | 0.173 | -2.533 | 3.3E-08 | 7.488 | 71984 | 11595 | 12439 | 7589 |
| LPE P-22:1(n-9) | C27H54NO6P | 518.3607 | 12.115 | 518.36160 | [M-H]- | Gut | 0.195 | -2.356 | 2.0E-07 | 6.706 | 94336 | 17762 | 18433 | 10263 |
| LPG 16:0 sn-1 | C22H45O9P | 483.2723 | 8.574 | 483.27284 | [M-H]- | Pancreas | 3.590 | 1.844 | 8.9E-03 | 2.051 | 47266 | 13260 | 169663 | 104153 |
| LPG 16:0 sn-2 | C22H45O9P | 483.2718 | 8.077 | 483.27284 | [M-H]- | Pancreas | 2.583 | 1.369 | 2.9E-04 | 3.534 | 9268 | 2270 | 23939 | 7607 |
| LPI 18:2(n-6,9) sn-1 | C27H49O12P | 595.2881 | 6.848 | 595.28889 | [M-H]- | Liver | 2.005 | 1.003 | 8.9E-06 | 5.049 | 41870 | 12834 | 83927 | 11979 |
| LPI 20:3 sn-1 | C29H51O12P | 621.3035 | 7.610 | 621.30454 | [M-H]- | Liver | 3.417 | 1.773 | 1.0E-05 | 4.992 | 24246 | 13490 | 82838 | 20752 |
| LPI 20:4(n-6,9,12,15) sn-2 | C29H49O12P | 619.2882 | 6.555 | 619.28889 | [M-H]- | Pancreas | 0.499 | -1.002 | 2.5E-03 | 2.597 | 62149 | 16781 | 31033 | 15541 |
| LPI 22:6 sn-1 | C31H49O12P | 643.2878 | 6.839 | 643.28889 | [M-H]- | Liver | 2.170 | 1.118 | 6.0E-05 | 4.222 | 19056 | 6665 | 41349 | 8953 |
| LPS 18:2 sn-1 | C24H44NO9P | 520.2677 | 6.775 | 520.26809 | [M-H]- | Liver | 2.517 | 1.331 | 9.9E-06 | 5.005 | 4154 | 1842 | 10453 | 1910 |
| MG 16:0(1) | C19H38O4 | 331.2842 | 10.639 | 331.28429 | [M+H]+ | Pancreas | 0.111 | -3.175 | 3.6E-03 | 2.440 | 118857 | 84428 | 13163 | 8546 |
| MG 16:0(2) | C19H38O4 | 331.2839 | 11.004 | 331.28429 | [M+H]+ | Pancreas | 0.124 | -3.010 | 4.5E-04 | 3.347 | 324529 | 170599 | 40285 | 29886 |
| MG 16:1(1) | C19H36O4 | 329.2684 | 8.977 | 329.26864 | [M+H]+ | Gut | 0.279 | -1.840 | 4.3E-03 | 2.367 | 27389 | 13685 | 7650 | 6788 |
| MG 16:1(1) | C19H36O4 | 329.2684 | 8.977 | 329.26864 | [M+H]+ | Pancreas | 0.030 | -5.039 | 1.6E-03 | 2.798 | 293995 | 203947 | 8941 | 7290 |
| MG 16:1(2) | C19H36O4 | 329.2687 | 9.271 | 329.26864 | [M+H]+ | Gut | 0.157 | -2.670 | 2.6E-03 | 2.592 | 216975 | 127241 | 34093 | 25129 |
| MG 16:1(2) | C19H36O4 | 329.2687 | 9.271 | 329.26864 | [M+H]+ | Pancreas | 0.029 | -5.111 | 1.5E-03 | 2.819 | 705145 | 486538 | 20406 | 20088 |
| MG 18:0(1) | C21H42O4 | 359.3157 | 13.043 | 359.31559 | [M+H]+ | Gut | 3.506 | 1.810 | 2.3E-03 | 2.640 | 7112 | 10912 | 24937 | 6393 |
| MG 18:0(1) | C21H42O4 | 359.3157 | 13.043 | 359.31559 | [M+H]+ | Pancreas | 0.250 | -2.002 | 4.2E-03 | 2.373 | 10964 | 6276 | 2737 | 2338 |
| MG 18:0(2) | C21H42O4 | 359.3156 | 13.472 | 359.31559 | [M+H]+ | Gut | 3.261 | 1.706 | 2.6E-03 | 2.588 | 59259 | 87977 | 193264 | 38497 |
| MG 18:1(1) | C21H40O4 | 357.3001 | 11.120 | 357.29994 | [M+H]+ | Pancreas | 0.075 | -3.736 | 5.0E-05 | 4.301 | 1413448 | 618499 | 106083 | 96957 |
| MG 18:1(2) | C21H40O4 | 357.3007 | 11.469 | 357.29994 | [M+H]+ | Pancreas | 0.102 | -3.296 | 7.0E-05 | 4.158 | 2466984 | 1063976 | 251250 | 258843 |
| MG 18:2(1) | C21H38O4 | 355.2843 | 9.612 | 355.28429 | [M+H]+ | Pancreas | 0.080 | -3.650 | 3.8E-04 | 3.416 | 811920 | 445014 | 64702 | 47330 |
| MG 18:2(2) | C21H38O4 | 355.2844 | 9.903 | 355.28429 | [M+H]+ | Pancreas | 0.088 | -3.509 | 6.6E-04 | 3.181 | 1449339 | 838222 | 127304 | 102891 |
| MG 18:3(1) | C21H36O4 | 353.2680 | 8.433 | 353.26864 | [M+H]+ | Pancreas | 0.043 | -4.543 | 2.4E-03 | 2.613 | 35408 | 25654 | 1519 | 1630 |
| MG 18:3(2) | C21H36O4 | 353.2684 | 8.684 | 353.26864 | [M+H]+ | Pancreas | 0.044 | -4.516 | 2.3E-03 | 2.648 | 107913 | 77318 | 4718 | 4195 |
| MG 20:3(2) | C23H40O4 | 381.2994 | 10.623 | 381.29994 | [M+H]+ | Pancreas | 0.244 | -2.037 | 1.3E-03 | 2.890 | 22482 | 11374 | 5479 | 3039 |
| MG 20:4(1) | C23H38O4 | 379.2844 | 9.512 | 379.28429 | [M+H]+ | Pancreas | 0.288 | -1.797 | 1.3E-03 | 2.889 | 30661 | 14575 | 8824 | 4022 |
| MG 20:4(2) | C23H38O4 | 379.2841 | 9.743 | 379.28429 | [M+H]+ | Pancreas | 0.439 | -1.189 | 6.4E-04 | 3.192 | 221244 | 55842 | 97053 | 51984 |
| MG 22:6(2) | C25H38O4 | 403.2840 | 9.499 | 403.28429 | [M+H]+ | Pancreas | 0.230 | -2.120 | 7.6E-03 | 2.117 | 145345 | 98471 | 33427 | 20783 |
| Oleic acid(n-9) | C18H34O2 | 283.2632 | 12.730 | 283.26316 | [M+H]+ | Gut | 0.497 | -1.009 | 1.0E-04 | 3.989 | 1443094 | 246883 | 716858 | 264717 |
| Oleic acid(n-9) | C18H34O2 | 283.2632 | 12.730 | 283.26316 | [M+H]+ | Pancreas | 0.414 | -1.272 | 3.5E-05 | 4.452 | 1122336 | 251611 | 464843 | 158659 |
| Oleic acid(n-9) | C18H34O2 | 281.2479 | 12.722 | 281.24860 | [M-H]- | Gut | 0.346 | -1.531 | 9.9E-05 | 4.003 | 23488 | 6313 | 8128 | 4028 |
| Oleic acid(n-9) | C18H34O2 | 281.2479 | 12.722 | 281.24860 | [M-H]- | Pancreas | 0.267 | -1.905 | 1.9E-04 | 3.732 | 14239 | 5230 | 3803 | 2232 |
| Oleic acid ethyl ester | C20H38O2 | 311.2944 | 18.044 | 311.29446 | [M+H]+ | Gut | 4.287 | 2.100 | 6.8E-03 | 2.167 | 4488 | 3347 | 19236 | 12547 |
| Oleic acid ethyl ester | C20H38O2 | 311.2944 | 18.044 | 311.29446 | [M+H]+ | Liver | 93.517 | 6.547 | 5.5E-04 | 3.261 | 1821 | 1360 | 170283 | 107036 |
| Oleic acid methyl ester (n-9) | C19H36O2 | 297.2797 | 16.664 | 297.27881 | [M+H]+ | Pancreas | 0.424 | -1.240 | 1.8E-03 | 2.743 | 2864719 | 880117 | 1213264 | 758862 |
| Palmitoleic acid methyl ester (n-7) | C17H32O2 | 269.2474 | 13.878 | 269.24751 | [M+H]+ | Pancreas | 0.098 | -3.358 | 5.9E-04 | 3.229 | 917062 | 517248 | 89460 | 67409 |
| PC 14:0_18:2(n-6,9) | C40H76NO8P | 730.5377 | 19.743 | 730.53813 | [M+H]+ | Gut | 4.193 | 2.068 | 1.5E-07 | 6.828 | 11868 | 7172 | 49756 | 7236 |
| PC 15:0_18:2(n-6,9) | C41H78NO8P | 744.5529 | 21.402 | 744.55378 | [M+H]+ | Gut | 5.515 | 2.463 | 2.0E-06 | 5.704 | 13176 | 5220 | 72668 | 20140 |
| PC 15:0_18:2(n-6,9) | C41H78NO8P | 788.5437 | 21.363 | 788.54471 | [M+HCOO-H]- | Gut | 5.081 | 2.345 | 2.6E-06 | 5.580 | 10835 | 4396 | 55057 | 15236 |
| PC 16:0_22:5(n-3,6,9,12,15) | C46H82NO8P | 852.5756 | 23.921 | 852.57601 | [M+HCOO-H]- | Liver | 0.131 | -2.932 | 3.7E-05 | 4.429 | 482006 | 199916 | 63151 | 7772 |
| PC 16:0_18:2(n-6,9) | C42H80NO8P | 802.5610 | 23.138 | 802.56036 | [M+HCOO-H]- | Gut | 3.257 | 1.703 | 4.2E-08 | 7.374 | 619110 | 166269 | 2016253 | 301855 |
| PC 16:0_18:3(n-6,9,12) | C42H78NO8P | 756.5533 | 21.282 | 756.55378 | [M+H]+ | Gut | 2.208 | 1.143 | 5.3E-07 | 6.280 | 19762 | 4782 | 43639 | 5376 |
| PC 16:0_18:3(n-6,9,12) | C42H78NO8P | 800.5433 | 21.245 | 800.54471 | [M+HCOO-H]- | Gut | 2.499 | 1.321 | 1.9E-08 | 7.722 | 12969 | 2855 | 32410 | 3373 |
| PC 16:0_20:5(n-3,6,9,12,15) | C44H78NO8P | 780.5532 | 20.311 | 780.55378 | [M+H]+ | Gut | 6.489 | 2.698 | 1.8E-07 | 6.734 | 6999 | 2320 | 45415 | 10658 |
| PC 16:0_20:5(n-3,6,9,12,15) | C44H78NO8P | 780.5532 | 20.311 | 780.55378 | [M+H]+ | Liver | 2.910 | 1.541 | 4.0E-05 | 4.397 | 93184 | 45949 | 271181 | 72269 |
| PC 16:0_20:5(n-3,6,9,12,15) | C44H78NO8P | 824.5436 | 20.275 | 824.54471 | [M+HCOO-H]- | Gut | 7.676 | 2.940 | 5.0E-07 | 6.301 | 4321 | 1586 | 33163 | 8802 |
| PC 16:0_20:5(n-3,6,9,12,15) | C44H78NO8P | 824.5436 | 20.275 | 824.54471 | [M+HCOO-H]- | Liver | 3.183 | 1.670 | 7.3E-05 | 4.139 | 55893 | 27290 | 177908 | 55976 |
| PC 16:0_22:4(n-6,9,12,15) | C46H84NO8P | 810.6003 | 25.247 | 810.60073 | [M+H]+ | Liver | 0.457 | -1.129 | 7.9E-04 | 3.104 | 121939 | 42810 | 55754 | 9768 |
| PC 16:0_22:5(n-3,6,9,12,15) | C46H82NO8P | 808.5842 | 23.968 | 808.58508 | [M+H]+ | Liver | 0.143 | -2.805 | 2.9E-05 | 4.539 | 837156 | 333831 | 119797 | 18041 |
| PC 16:1_18:1 | C42H80NO8P | 758.5691 | 22.817 | 758.56943 | [M+H]+ | Gut | 2.429 | 1.280 | 1.4E-05 | 4.861 | 32766 | 8704 | 79587 | 17370 |
| PC 16:1(n-7)_18:2(n-6,9) | C42H78NO8P | 756.5530 | 20.130 | 756.55378 | [M+H]+ | Gut | 3.025 | 1.597 | 2.6E-05 | 4.581 | 11223 | 4263 | 33949 | 9117 |
| PC 16:1(n-7)_18:2(n-6,9) | C42H78NO8P | 800.5440 | 20.096 | 800.54471 | [M+HCOO-H]- | Gut | 3.144 | 1.653 | 1.5E-05 | 4.835 | 8724 | 3205 | 27427 | 7139 |
| PC 16:1(n-7)_20:4(n-6,9,12,15) | C44H78NO8P | 780.5531 | 19.655 | 780.55378 | [M+H]+ | Liver | 0.281 | -1.831 | 6.3E-06 | 5.200 | 460922 | 126621 | 129532 | 43948 |
| PC 16:1(n-7)_20:4(n-6,9,12,15) | C44H78NO8P | 780.5531 | 19.655 | 780.55378 | [M+H]+ | Pancreas | 0.369 | -1.438 | 6.3E-03 | 2.202 | 8818 | 4130 | 3254 | 2379 |
| PC 16:1(n-7)_20:4(n-6,9,12,15) | C44H78NO8P | 824.5438 | 19.620 | 824.54471 | [M+HCOO-H]- | Liver | 0.275 | -1.864 | 1.1E-05 | 4.947 | 340354 | 99986 | 93481 | 32992 |
| PC 16:1(n-7)_20:4(n-6,9,12,15) | C44H78NO8P | 824.5438 | 19.620 | 824.54471 | [M+HCOO-H]- | Pancreas | 0.339 | -1.562 | 1.6E-03 | 2.802 | 6329 | 2523 | 2143 | 1491 |
| PC 16:1(n-7)_22:6(n-3,6,9,12,15,18) | C46H78NO8P | 804.5532 | 18.892 | 804.55378 | [M+H]+ | Liver | 0.354 | -1.500 | 8.0E-04 | 3.099 | 273432 | 110181 | 96708 | 40572 |
| PC 16:1(n-7)_22:6(n-3,6,9,12,15,18) | C46H78NO8P | 848.5434 | 18.858 | 848.54471 | [M+HCOO-H]- | Liver | 0.344 | -1.538 | 3.8E-04 | 3.425 | 204058 | 76145 | 70277 | 28783 |
| PC 17:0_18:2 | C43H82NO8P | 816.5755 | 25.075 | 816.57601 | [M+HCOO-H]- | Gut | 6.023 | 2.591 | 2.3E-06 | 5.637 | 8410 | 2609 | 50659 | 14795 |
| PC 17:0_18:2 | C43H82NO8P | 816.5755 | 25.075 | 816.57601 | [M+HCOO-H]- | Liver | 2.923 | 1.548 | 2.3E-05 | 4.644 | 43091 | 13652 | 125966 | 35184 |
| PC 17:0_18:2 | C43H82NO8P | 772.5848 | 25.122 | 772.58508 | [M+H]+ | Gut | 5.054 | 2.338 | 2.0E-06 | 5.690 | 14331 | 5728 | 72431 | 19529 |
| PC 17:0_18:2 | C43H82NO8P | 772.5848 | 25.122 | 772.58508 | [M+H]+ | Liver | 2.793 | 1.482 | 2.6E-06 | 5.592 | 73636 | 21031 | 205639 | 44556 |
| PC 18:0_18:2(n-6,9) | C44H84NO8P | 786.6015 | 26.972 | 786.60073 | [M+H]+ | Gut | 2.613 | 1.386 | 1.2E-06 | 5.924 | 362534 | 106735 | 947371 | 158998 |
| PC 18:0_18:2(n-6,9) | C44H84NO8P | 786.6015 | 26.972 | 786.60073 | [M+H]+ | Liver | 2.808 | 1.490 | 7.9E-08 | 7.105 | 2025867 | 528197 | 5688297 | 874349 |
| PC 18:0_18:2(n-6,9) | C44H84NO8P | 830.5915 | 26.942 | 830.59166 | [M+HCOO-H]- | Gut | 2.743 | 1.456 | 9.0E-07 | 6.044 | 280754 | 79468 | 770164 | 135430 |
| PC 18:0_18:2(n-6,9) | C44H84NO8P | 830.5915 | 26.942 | 830.59166 | [M+HCOO-H]- | Liver | 2.677 | 1.421 | 5.6E-07 | 6.252 | 1568320 | 463040 | 4198873 | 727188 |
| PC 18:1(n-9)_18:2(n-6,9) | C44H82NO8P | 784.5851 | 23.513 | 784.58508 | [M+H]+ | Gut | 3.260 | 1.705 | 2.0E-06 | 5.708 | 181877 | 59429 | 592963 | 129354 |
| PC 18:1(n-9)_18:2(n-6,9) | C44H82NO8P | 784.5850 | 24.616 | 784.58508 | [M+H]+ | Gut | 3.001 | 1.586 | 1.5E-05 | 4.811 | 4694 | 2437 | 14086 | 3020 |
| PC 18:1(n-9)_18:2(n-6,9) | C44H82NO8P | 828.5753 | 23.471 | 828.57601 | [M+HCOO-H]- | Gut | 3.367 | 1.752 | 2.0E-06 | 5.697 | 121337 | 38859 | 408543 | 91993 |
| PC 18:1_20:3 | C46H84NO8P | 810.5996 | 24.472 | 810.60073 | [M+H]+ | Gut | 2.266 | 1.180 | 9.7E-04 | 3.014 | 32602 | 16283 | 73890 | 21405 |
| PC 18:1_20:3 | C46H84NO8P | 854.5918 | 24.427 | 854.59166 | [M+HCOO-H]- | Gut | 2.768 | 1.469 | 5.8E-05 | 4.235 | 16775 | 6255 | 46437 | 12780 |
| PC 18:1(n-9)_20:4(n-6,9,12,15) | C46H82NO8P | 808.5854 | 22.883 | 808.58508 | [M+H]+ | Liver | 0.447 | -1.161 | 3.4E-05 | 4.467 | 3734125 | 931733 | 1670038 | 295435 |
| PC 18:1(n-9)_20:4(n-6,9,12,15) | C46H82NO8P | 852.5756 | 22.841 | 852.57601 | [M+HCOO-H]- | Liver | 0.454 | -1.138 | 2.5E-04 | 3.607 | 2100253 | 636977 | 954546 | 191816 |
| PC 18:2(n-6,9)/18:2(n-6,9) | C44H80NO8P | 782.5686 | 20.754 | 782.56943 | [M+H]+ | Gut | 5.072 | 2.343 | 8.9E-08 | 7.052 | 21640 | 8694 | 109758 | 21640 |
| PC 18:2(n-6,9)/18:2(n-6,9) | C44H80NO8P | 826.5599 | 20.717 | 826.56036 | [M+HCOO-H]- | Gut | 5.235 | 2.388 | 9.2E-08 | 7.038 | 15287 | 5627 | 80021 | 16281 |
| PC 18:2(n-6,9)_20:4(n-6,9,12,15) | C46H80NO8P | 806.5691 | 20.256 | 806.56943 | [M+H]+ | Gut | 2.087 | 1.061 | 1.8E-05 | 4.734 | 34609 | 11598 | 72214 | 10462 |
| PC 18:2(n-6,9)_20:4(n-6,9,12,15) | C46H80NO8P | 850.5595 | 20.220 | 850.56036 | [M+HCOO-H]- | Gut | 2.249 | 1.169 | 1.1E-05 | 4.978 | 23526 | 7842 | 52901 | 8603 |
| PC 20:4(n-6,9,12,15)_22:6(n3,6,9,12,15,18) | C50H80NO8P | 854.5681 | 18.892 | 854.56943 | [M+H]+ | Liver | 0.488 | -1.034 | 1.5E-03 | 2.814 | 204761 | 70272 | 100012 | 27748 |
| PC 20:4(n-6,9,12,15)_22:6(n3,6,9,12,15,18) | C50H80NO8P | 898.5590 | 18.860 | 898.56036 | [M+HCOO-H]- | Liver | 0.457 | -1.129 | 1.1E-03 | 2.950 | 154179 | 54162 | 70483 | 20732 |
| PC 36:2(n-6,9) | C44H84NO8P | 786.5997 | 26.672 | 786.60073 | [M+H]+ | Gut | 2.243 | 1.165 | 9.3E-06 | 5.031 | 225498 | 61825 | 505773 | 92197 |
| PC 36:2(n-6,9) | C44H84NO8P | 830.5913 | 26.635 | 830.59166 | [M+HCOO-H]- | Gut | 2.453 | 1.294 | 2.5E-05 | 4.594 | 153054 | 44860 | 375417 | 87057 |
| PC P-16:0 / 18:2(n-6,9) | C42H80NO7P | 742.5741 | 24.326 | 742.57452 | [M+H]+ | Gut | 4.136 | 2.048 | 2.6E-09 | 8.589 | 30839 | 10604 | 127546 | 15522 |
| PC P-16:0 / 18:2(n-6,9) | C42H80NO7P | 786.5645 | 24.279 | 786.56544 | [M+HCOO-H]- | Gut | 4.408 | 2.140 | 6.5E-10 | 9.186 | 19383 | 4649 | 85432 | 10664 |
| PE 16:0_22:5(n-6,9,12,15,18) | C43H76NO8P | 766.5369 | 24.214 | 766.53813 | [M+H]+ | Gut | 0.433 | -1.209 | 1.2E-03 | 2.923 | 18589 | 6032 | 8041 | 3231 |
| PE 16:0_22:5(n-6,9,12,15,18) | C43H76NO8P | 766.5369 | 24.214 | 766.53813 | [M+H]+ | Liver | 0.187 | -2.421 | 1.4E-03 | 2.869 | 172751 | 99455 | 32267 | 6747 |
| PE 16:0_22:5(n-6,9,12,15,18) | C43H76NO8P | 764.5230 | 24.164 | 764.52358 | [M-H]- | Gut | 0.480 | -1.060 | 1.0E-03 | 2.996 | 24861 | 7175 | 11922 | 4017 |
| PE 16:0_22:5(n-6,9,12,15,18) | C43H76NO8P | 764.5230 | 24.164 | 764.52358 | [M-H]- | Liver | 0.169 | -2.567 | 2.1E-03 | 2.685 | 199044 | 123990 | 33591 | 5243 |
| PE 16:1_20:4 | C41H72NO8P | 736.4911 | 19.767 | 736.49228 | [M-H]- | Liver | 0.410 | -1.285 | 3.3E-05 | 4.481 | 79389 | 18580 | 32576 | 11967 |
| PE 16:1_20:4 | C41H72NO8P | 738.5053 | 19.800 | 738.50683 | [M+H]+ | Liver | 0.447 | -1.161 | 3.2E-05 | 4.497 | 58700 | 12264 | 26257 | 9081 |
| PE 18:0_18:2(n-6,9) | C41H78NO8P | 742.5396 | 27.232 | 742.53923 | [M-H]- | Gut | 2.040 | 1.029 | 6.4E-04 | 3.191 | 65433 | 18630 | 133489 | 38471 |
| PE 18:0_22:6(n-3,6,9,12,15,18) | C45H78NO8P | 792.5531 | 24.556 | 792.55378 | [M+H]+ | Liver | 0.299 | -1.742 | 4.3E-03 | 2.371 | 54089 | 29936 | 16175 | 9730 |
| PE 18:1(n-9)_18:2(n-6,9) | C41H76NO8P | 742.5376 | 23.761 | 742.53813 | [M+H]+ | Gut | 2.013 | 1.009 | 1.7E-04 | 3.779 | 35183 | 10332 | 70806 | 15897 |
| PE 18:1(n-9)_18:2(n-6,9) | C41H76NO8P | 742.5376 | 23.761 | 742.53813 | [M+H]+ | Liver | 2.492 | 1.317 | 1.0E-05 | 4.999 | 230249 | 33408 | 573776 | 141015 |
| PE 18:1(n-9)_18:2(n-6,9) | C41H76NO8P | 740.5228 | 23.715 | 740.52358 | [M-H]- | Liver | 2.627 | 1.394 | 4.5E-05 | 4.347 | 252965 | 41564 | 664631 | 195932 |
| PE 18:2/18:2 | C41H74NO8P | 738.5075 | 20.887 | 738.50793 | [M-H]- | Gut | 2.771 | 1.470 | 2.2E-05 | 4.657 | 3408 | 1421 | 9443 | 2179 |
| PE 18:2/18:2 | C41H74NO8P | 738.5075 | 20.887 | 738.50793 | [M-H]- | Liver | 3.022 | 1.596 | 4.3E-05 | 4.366 | 25919 | 5121 | 78336 | 24870 |
| PE P-16:0_20:5(n-3,6,9,12,15) | C41H72NO7P | 722.5123 | 21.445 | 722.51192 | [M+H]+ | Gut | 3.306 | 1.725 | 2.3E-06 | 5.633 | 10577 | 2564 | 34962 | 8249 |
| PE P-16:0_20:5(n-3,6,9,12,15) | C41H72NO7P | 720.4961 | 21.405 | 720.49736 | [M-H]- | Gut | 3.466 | 1.793 | 3.0E-05 | 4.527 | 12080 | 2471 | 41871 | 13290 |
